# A Multi-Target Pharmacological Correction of a Lipoyltransferase *LIPT1* Gene Mutation in Patient-Derived Cellular Models

**DOI:** 10.3390/antiox13081023

**Published:** 2024-08-22

**Authors:** David Gómez-Fernández, Ana Romero-González, Juan M. Suárez-Rivero, Paula Cilleros-Holgado, Mónica Álvarez-Córdoba, Rocío Piñero-Pérez, José Manuel Romero-Domínguez, Diana Reche-López, Alejandra López-Cabrera, Salvador Ibáñez-Mico, Marta Castro de Oliveira, Andrés Rodríguez-Sacristán, Susana González-Granero, José Manuel García-Verdugo, José A. Sánchez-Alcázar

**Affiliations:** 1 Centro Andaluz de Biología del Desarrollo (CABD-CSIC-Universidad Pablo de Olavide), 41013 Sevilla, Spain; dgomfer1@acu.upo.es (D.G.-F.); aromgon1@upo.es (A.R.-G.); jmsuariv@upo.es (J.M.S.-R.); pcilhol@upo.es (P.C.-H.); malvcor@upo.es (M.Á.-C.); rpieper@alu.upo.es (R.P.-P.); jmromdom@upo.es (J.M.R.-D.); dreclop@alu.upo.es (D.R.-L.); alopcab2@alu.upo.es (A.L.-C.); 2 Hospital Clínico Universitario Virgen de la Arrixaca, Servicio de Neuropediatría, 30120 Murcia, Spain; salvador.ibanez@carm.es; 3 Neuropediatria, Neurolinkia, C. Jardín de la Isla, 8, Local 4 y 5, 41014 Sevilla, Spain; martadecastro@neurolinkia.com; 4 FEA Pediatría, Centro Universitario Hospitalar de Faro, R. Leão Penedo, 8000-386 Faro, Portugal; 5 Neuropediatria, Servicio de Pediatría, Hospital Universitario Virgen Macarena, 41009 Sevilla, Spain; arodriguezsacristan@us.es; 6 Departamento de Farmacología, Radiología y Pediatría de la Facultad de Medicina de la Universidad de Sevilla, 41009 Sevilla, Spain; 7 Laboratory of Comparative Neurobiology, Cavanilles Institute of Biodiversity and Evolutionary Biology, University of Valencia and CIBERNED-ISCIII, 46980 Valencia, Spain; susana.gonzalez@uv.es (S.G.-G.); j.manuel.garcia@uv.es (J.M.G.-V.)

**Keywords:** LIPT1, SIRT3, fibroblasts, lipoylation, bioenergetics, 2-ketoacid dehydrogenase

## Abstract

Mutations in the *lipoyltransferase 1* (*LIPT1*) gene are rare inborn errors of metabolism leading to a fatal condition characterized by lipoylation defects of the 2-ketoacid dehydrogenase complexes causing early-onset seizures, psychomotor retardation, abnormal muscle tone, severe lactic acidosis, and increased urine lactate, ketoglutarate, and 2-oxoacid levels. In this article, we characterized the disease pathophysiology using fibroblasts and induced neurons derived from a patient bearing a compound heterozygous mutation in *LIPT1.* A Western blot analysis revealed a reduced expression of LIPT1 and absent expression of lipoylated pyruvate dehydrogenase E2 (PDH E2) and alpha-ketoglutarate dehydrogenase E2 (α-KGDH E2) subunits. Accordingly, activities of PDH and α-KGDH were markedly reduced, associated with cell bioenergetics failure, iron accumulation, and lipid peroxidation. In addition, using a pharmacological screening, we identified a cocktail of antioxidants and mitochondrial boosting agents consisting of pantothenate, nicotinamide, vitamin E, thiamine, biotin, and α-lipoic acid, which is capable of rescuing *LIPT1* pathophysiology, increasing the LIPT1 expression and lipoylation of mitochondrial proteins, improving cell bioenergetics, and eliminating iron overload and lipid peroxidation. Furthermore, our data suggest that the beneficial effect of the treatment is mainly mediated by SIRT3 activation. In conclusion, we have identified a promising therapeutic approach for correcting *LIPT1* mutations.

## 1. Introduction

In the 1950s, Jukes et al. and Reed et al. discovered α-lipoic acid (α-LA), an essential cofactor for mitochondrial function [1,2]. α-LA or 6,8-dithiooctanoic acid is covalently bound to the ε-amino group of lysine residues and functions as a cofactor for the activity of essential mitochondrial enzymes [3], including pyruvate dehydrogenase (PDH), alpha-ketoglutarate dehydrogenase (α-KGDH), 2-oxoadipate dehydrogenase (OADH), branched chain ketoacid dehydrogenase (BCKDH), and the glycine cleavage system (GCS) [2,4,5]. A certain number of proteins are necessary to the biosynthesis of lipoic acid, and mutations in several genes are known to cause human mitochondrial diseases [4].

α-LA possesses a disulfide bond that provides a source of reductive potential required for the catalysis by mitochondrial dehydrogenases and participates in the stabilization and redox-dependent regulation of these multienzyme complexes [6]. These functions make lipoic acid essential for cell growth, the oxidation of energy sources, glycine degradation, and the regulation of mitochondrial redox balance [7]. α-LA metabolism has been thoroughly studied in prokaryotes [8] and yeast [9], but is less well understood in superior organisms. In mammals, the α-LA biosynthetic pathway is carried out by the lipoyl (octanoyl) transferase 2 (LIPT2) and the lipoic acid synthase (LIAS). In addition, LIPT1 allows the lipoylation of several enzymes [7]. LIPT2 transfers octanoate from the acyl carrier protein (ACP) to the glycine cleavage system H protein (GCSH). Then, LIAS inserts sulfur atoms into the octanoyl group on GCSH, while LIPT1 transfers the lipoyl group from the GCSH to E2 dehydrogenases’ protein subunits. Deficiencies in either of these enzymes, as well as disruptions in mitochondrial fatty acid synthesis type II (mtFASII), ACP, or iron–sulfur cluster biogenesis, result in a diminished lipoylation of PDH or α-KGDH, leading to impaired mitochondrial function [4].

One key difference in α-LA metabolism between *Escherichia coli* and *Homo sapiens* is the versatility of the bacterial enzyme lipoate-protein ligase A (LplA), , which is able to conjugate not only endogenous α-LA but also exogenous α-LA to an adenylate intermediate, lipoyl-adenosin monophosphate or lipoyl-AMP, followed by ligation to the lipoyl domain of E2 subunits and GCSH. Also, LplA could use both α-LA and octanoate to modify E2 subunits [10]. In contrast, human LIPT1 is only able to use endogenous α-LA, although a report identified a mammalian lipoic acid-activating enzyme, known as a acyl-Coenzyme A (CoA) synthethase medium-chain family member 2A (ACSM2A), that could activate exogenous lipoic acid with GTP [11]; however, there has been no substantial evidence to support that this enzyme functions in α-LA metabolism in vivo.

Pathologies related with α-LA are considered inborn errors of metabolism (IEMs), which are genetic disorders resulting from an enzyme defect in biochemical and metabolic pathways [12]. *LIPT1* mutations cause mitochondrial diseases including the Leigh syndrome variants [13]. Mutations in α-LA metabolism are characterized by lactic acidosis, epilepsy, developmental delay, Leigh-like encephalopathy, and early death [14,15]. In contrast to *LIAS* or *LIPT2* mutations, glycine cleavage is normal in most mutant *LIPT1* patients and there are normal glycine serum levels [15]. This lack of glycine elevation suggests sparing of the GCS, consistent with the fact that this enzymatic complex does not depend on LIPT1 for lipoylation.

Currently, there is no treatment for LIPT1 deficiency in humans. In yeast, the depletion of *LIPT1* ortholog lipoate-protein ligase 3 (*lip3*) showed a growth defect that could be rescued by α-LA supplementation, while human fibroblasts showed only a moderate increase in PDH activity but not in α-KGDH [16]. Genetic therapy has been proposed as inserting the bacterial ligase, LplA, into the mitochondria or the nuclear genome [14]. In fact, the *E. coli* lipoate ligase is known to modify human lipoylated enzymes [17]. However, there is still a long way to go until gene therapy is a reality for patient treatment.

In this work, we evaluated the positive effect of a cocktail of antioxidants and mitochondrial activators on the mutant phenotype of fibroblasts and iNs derived from a patient with a compound heterozygous mutation in the *LIPT1* gene.

## 2. Materials and Methods

### 2.1. Reagents

Anti-mitochondrially encoded Cytocrome C Oxidase Subunit II (mt-CO2) (ab79393), anti-voltage-dependent anion channel 1 (VDAC1) (ab14734), anti-ATP-synthase F1 subunit 1 alpha (ATP5F1A) (ab14748), anti-NADH:Ubiquinone Oxidoreductase Subunit A9 (NDUFA9) (ab14713), anti-Activating Transcription Factor 5 (ATF5) (ab184923), anti-Lon peptidase 1 (Lonp1) (ab103809), anti-sirtuin 1 (SIRT1) (ab110304), anti-nuclear respiratory factor 2 (Nrf2) (ab62352), anti-peroxisome proliferator-activated receptor γ coactivator 1α (PGC1α) (ab191838), anti-manganese superoxide dismutase (MnSOD) (ab68155), anti-pyruvate dehydrogenase subunit E2 (PDH E2) (ab110332), anti-Cytochrome C Oxidase Subunit IV (COX IV) (ab14744), Goat anti-Rabbit IgG H&L (HRP) (ab6721), Rabbit anti-Mouse IgG H&L (HRP) (ab6728), and Rabbit anti-Goat IgG H&L (HRP) (ab6741) were purchased from Abcam (Cambridge, UK).

Anti-nuclear respiratory factor 1 (Nrf1) (NBP1-778220) was purchased from Novus Biologicals ( Madrid, Spain). Anti-actin (MBS448085) and anti-mitochondrially encoded NADH:ubiquinone oxidoreductase core subunit 6 (mt-ND6) (MBS8518686) were purchased from MyBioSource (San Diego, CA, USA). Anti-Ubiquinol Cytochrome C Reductase Core Protein 1 (UQCRC1) (459140), anti-lipoyltransferase 1 (LIPT1) (PA5-57064), anti-heat shock protein 60 (hsp60) (MA3-012), anti-heat shock protein 70 (hsp70) (MA3-028), anti-sirtuin 3 (SIRT3) (PA5-13222), anti-translocase of the outer mitochondrial membrane 20 (TOMM20) (H00009804-M01), and nicotinamide (A15970.30) were purchased from ThermoFisher Scientific (Waltham, MA, USA). Anti-PGC-1α (4C1.3) and anti-lipoic acid (LA) (437695) were purchased from Merck Millipore (Burlington, MA, USA).

Anti-ketoglutarate dehydrogenase subunit E2 (KGDH E2) (26865S), anti-Activating Transcription Factor 4 (ATF4) (11815S), and anti-mitochondrial transcription factor A (TFAM) (7495S) were purchased from Cell Signaling (Danvers, MA, USA). Anti-phosphorylated PGC1α (P-PGC1α) (AF6650) was purchased from R&D Systems (Minneapolis, MN, USA). Anti-Forkhead Box O3 (FOXO3A) (sc-48348), anti-LA (sc-101354), anti-Tau (sc-21796), D-galactose (sc-202564), Deferiprone (sc-211220), rotenone (sc-203342), paraformaldehyde (PFA) (sc-253236B), oligomycin (sc-203342), antimycin A (sc-202467A), carbonyl cyanide 4-(trifluoromethoxy)phenylhydrazone (FCCP) (sc-203578), 6,8-bis (benzylthiol)-octanoic acid (CPI-613) (sc-482709), thiamine (sc-205859), biotin (sc-20476), and 4-(2-hydroxyethyl)-1-piperazine ethanesulfonic acid (HEPES) (sc-29097) were purchased from Santa Cruz Biotechnology (Dallas, TX, USA).

Prussian Blue (03899), Sudan Black (199664), glutaraldehyde 25% Aqueous Solution (G5882), Luperox^®^ DI, *tert*-butyl peroxide (168521), α-LA (62320), α-Tocopherol/Vitamin E (T3251), dimethyl sulfoxide (DMSO) (17093), and donkey serum (D9663) were purchased from Sigma-Aldrich (Saint Louis, MO, USA). Sodium pantothenate (17228) was purchased from Cayman Chemical (Ann Harbor, MI, USA). Mitotracker^TM^ Red CMXRos (M46752), Bovine Serum Albumine (BSA) (BP9702-100), Hoescht (10150888), and 4′,6-diamidino-2-phenylindole (DAPI) were purchased from Invitrogen^TM^/Molecular Probes (Eugene, OR, USA). Phosphate-buffered saline (PBS) (102309) was purchased from Intron Biotechnology (Seongnam, Republic of Korea). 3-(1H-1,2,3-triazol-4-yl) pyridine (3-TYP) (HY-108331) was purchased from MedChemExpress (Sollentuna, Sweden).

### 2.2. Ethical Statements

Approval of the ethical committee of the Hospital Universitario Virgen Macarena y Virgen del Rocío in Sevilla (Spain) was obtained, according to the principles of the Declaration of Helsinki as well as the International Conferences on Harmonization and Good Clinical Practice Guidelines.

### 2.3. Fibroblast Culture

Cultured fibroblasts were derived from a skin biopsy of one 7-year-old boy patient with the following compound heterozygous mutation in the *LIPT1* gene (NM_001204830), c.212C>T (p.Ser71Phe) and c.292C>T (p.Arg98Trp), previously reported as pathogenic variants [13]. Control fibroblasts were human skin primary fibroblasts from two healthy volunteer donors. These control cells were sex- and age-matched. Samples from the patient and controls were obtained according to the Helsinki Declarations of 1964, as revised in 2001. Fibroblasts derived from the patient and controls were cultured at 37 °C and 5% CO_2_ in Dulbecco’s Modified Eagle Medium (DMEM)containing 4.5 g glucose/L, L-glutamine, and pyruvate supplemented with a 1% antibiotic Pen-Strep solution (Sigma-Aldrich, Saint Louis, MO, USA) and 10–20% Fetal Bovine Serum (FBS) (Gibco^TM^, Waltham, MA, USA). All the experiments were performed with fibroblasts on a passage number lower than 8.

### 2.4. Drug Screening

Drug screening was performed in a restrictive culture medium with galactose as the main carbon source. Our aim was to deprive cells from glycolysis as an energy source (due to the use of galactose) and hence have them rely exclusively on the oxidative phosphorylation (OXPHOS) for ATP production [18,19]. In these cell culture conditions, mutant *LIPT1* fibroblasts were unable to survive.

The galactose medium was prepared with DMEM without glucose and glutamine (Invitrogen^TM^/Molecular Probes, Eugene, OR, USA) supplemented with 10 mM D-galactose, 10 mM HEPES, a 1% antibiotic Pen-Strep solution, and 10% FBS. Cells were seeded in 24-well plates in DMEM containing 1 g glucose/L. After 24 h, cells were treated with different compounds for seven days. Next, the medium was removed, and cells were washed twice with PBS prior to the addition of the galactose medium. Then, the treatments were re-applied in the same concentration and images were taken in 24 h intervals for 72 h. Cell counting and representative images were obtained immediately (T0) and 72 h after the shift to the galactose medium, using the BioTek™ Cytation™ 1 Cell Imaging Multi-Mode Reader (Biotek, Winooski, VT, USA). The proliferation ratio was obtained by dividing the number of cells at T72 by the number of cells at T0. Proliferation ratio values above 1 were considered as cell proliferation, while values below 1 were considered as cell death, and a value of 1 indicated cell survival. Compounds considered positive allowed the survival of mutant cells in the galactose medium. Cell viability was confirmed by trypan blue dye exclusion.

The same screening was repeated using 3-TYP, a SIRT3-specific inhibitor. To ensure the specific inhibition of this SIRT3, the concentration selected was 50 nM, as this compound exhibits an IC_50_ (half-maximal inhibitory concentration) of 16 nM for SIRT3. The IC_50_ for SIRT1 and SIRT2 are 88 nM and 92 nM, respectively, requiring a higher concentration of 3-TYP to inhibit these sirtuins. The procedure is similar; cells are seeded in a glucose medium and treated for 3 days. Then, the glucose medium is replaced with a galactose medium and when the treatment is renewed, we add 3-TYP for the last 72 h. The images were taken and analyzed as previously described.

### 2.5. Quantitative Real-Time PCR (qPCR)

The expression levels of the *LIPT1* gene were assessed by qPCR in untreated and treated mutant fibroblasts as well as in control cells, using mRNA extracts. Total RNA extraction was carried out using the RNeasy Mini Kit (74104, Qiagen, Venlo, the Netherlands). cDNA synthesis from 1 µg of RNA was performed by the iScript cDNA KIT (170-8891, BioRad, Hercules, CA, USA). Consequently, qPCR was conducted following standard procedures and the SYBR Green Protocol. *LIPT1* primers: 5′-CTG AAT CTC GCT CTG TTG CC-3′ (FW) and 5′-TGG GAC CTG GCA GTT ACA AA-3′ (RV). *Actin* was used as a housekeeping control gene and the primers utilized were 5′-AGAGCTACGAGCTGCCTGAC-3′ (FW) and 3′-AGCACTGTGTTGGCGTACAG-5′ (RV). Primer design was facilitated using the online tool Primer3 (https://primer3.ut.ee/, accessed on 18 March 2023).

### 2.6. Immunoblotting

A Western blotting assay was performed using standard methods. After transferring the proteins to nitrocellulose membranes (1620115, Bio-Rad, Hercules, CA, USA), these were blocked in BSA 5% in TTBS (blocking solution) for 1 h and then incubated with primary antibodies, which were diluted 1:1000 in the blocking solution overnight at 4 °C. Then, membranes were washed twice with TTBS and incubated with the corresponding secondary antibody coupled to horseradish peroxidase (HRP) (1:2500 dilution in BSA 5%) for 1 h. Protein loading was checked for every membrane using Ponceau S staining and actin protein levels. ChemiDoc™ MP Imaging System (BioRad, Hercules, CA, USA) was used to reveal protein signals. The results obtained were normalized to the mean expression levels of control cells and the actin protein.

If possible, when the molecular weight of new proteins of interest did not interfere, membranes were re-probed with different antibodies. In the case of proteins with a different molecular weight, membranes were cut and detected with different antibodies. Results were analyzed by ImageLab™ software version 6.1. (BioRad, Hercules, CA, USA).

### 2.7. Prussian Blue Staining

Iron accumulation was determined by Perl’s Prussian Blue (PPB) staining in control and patient-derived fibroblasts and induced neurons (iNs) [20]. Images were taken by a light and fluorescence Axio Vert A1 microscope (Zeiss, Oberkochen, Germany) with a 20× objective and analyzed by Fiji-ImageJ software version 2.9.0. Moreover, iron content was measured in cell culture extracts by inductively coupled plasma mass spectrometry (ICP-MS) [21]. ICP-MS was performed with an *Agilent* 7800 spectrometer (Agilent Technologies, Santa Clara, CA, USA). Cell extracts were obtained by acid digestion with HNO_3_.

### 2.8. Sudan Black Staining

Lipofuscin accumulation was assessed by Sudan Black staining in control and patient-derived fibroblasts as previously described [22,23]. Images were taken by a light and fluorescence Axio Vert A1 microscope (Zeiss, Oberkochen, Germany) with a 20× objective and analyzed by Fiji-ImageJ software version 2.9.0.

### 2.9. TEM Analysis

The cells were seeded on 8-well Permanox chamber slides (Nunc, ThermoFisher Scientific, Waltham, MA, USA). They were washed three times with a 0.1 M phosphate buffer (PB). Then, cells can be fixed in tempered 3.5% glutaraldehyde in 0.1 M PB for 5 min at 37 °C or for 55 min at 4 °C. Cells were postfixed in 2% OsO_4_ for 1 h at room temperature, rinsed, dehydrated, and embedded in Durcupan resin (Sigma-Aldrich, Saint Louis, MO, USA). Later, ultra-thin (70 nm) sections of the cells were cut with a diamond knife and examined by a transmission electron microscope (TEM) (FEI Tecnai G2 Spirit BioTwin) with a Xarosa (20-Megapixel resolution) digital camera using Radius image acquisition software version 2.1. (EMSIS GmbH, Münster, Germany).

### 2.10. PDH and KGDH Activities

PDH and KGDH activities were assessed according to the protocols established by PDH Enzyme Activity Dipstick Assay Kit (ab109882) and α-Ketoglutarate Dehydrogenase Activity Assay Kit (ab185440). Signal intensity was acquired using the Chemidoc^TM^ MP Imaging System and analyzed using ImageLab^TM^ software version 6.1. (BioRad, Hercules, CA, USA).

### 2.11. Immunofluorescence Microscopy

Cells were seeded on 1 mm width glass coverslips (631-1331, Menzel-Gläser, ThermoFisher Scientific, Waltham, MA, USA) for 72 h in the DMEM glucose medium with/without the addition of CocT. Then, they were washed twice with PBS 1x and fixed in 4% PFA for 10 min at room temperature. Cells were incubated in a blocking buffer (1% BSA in PBS) for 30 min and permeabilized with 0.1% saponin in the blocking buffer for 15 min. In the meantime, primary antibodies were diluted 1:100 in an antibody buffer (0.5% BSA and 0.1% saponin in PBS) and then incubated overnight at 4 °C. Following primary antibodies’ incubation, cells were washed twice with PBS 1x, and secondary antibodies were similarly diluted 1:400 in the antibody buffer. Cells were incubated for 2 h at room temperature. Subsequently, after two washes with PBS 1x, they were incubated for 5 min with 1 µg/mL of DAPI and washed again with PBS 1x. Finally, coverslips were mounted on microscope slides using 10 µL of Mowiol.

Images were taken using a DeltaVision system (Applied Precision; Issaquah, WA, USA) with an Olympus IX-71 microscope using a 40x objective. They were analyzed using the softWoRx and Fiji-ImageJ software version 2.9.0. The microscope settings were consistently maintained across each experiment.

### 2.12. Measurement of Membrane Potential

The measurement of mitochondrial membrane potential was conducted using Mitotracker^TM^ Red CMXRos, a fluorescent dye sensitive to mitochondrial membrane potential. Untreated and treated cells were seeded on 1 mm glass coverslips in the DMEM glucose medium for three days. Subsequently, cells were stained with 100 nM Mitotracker^TM^ Red CMXRos for 45 min at 37 °C before fixation. Once cells were stained, we proceeded with two washes with PBS 1x, and they were fixed with 4% PFA for 10 min. Then, we incubated the cells with 1 µg/mL of DAPI for 10 min. Finally, after 5 washes with PBS 1x, we mounted the coverslips on microscope slides with 10 µL of Mowiol. Images were obtained using a DeltaVision system (Applied Precision; Issaqua, WA, USA) with an Olympus IX-71 fluorescent microscope with a 40x objective and they were analyzed using Fiji-ImageJ software version 2.9.0. The mitochondrial membrane potential was calculated based on fluorescence intensity. The microscope settings were consistently maintained in each experiment.

### 2.13. Bioenergetics

The mitochondrial respiratory function of control and mutant fibroblasts was measured using a mitostress test assay with an XFe24 extracellular flux analyzer (Seahorse Bioscience, Billerica, MA, USA, 102340-100) according to the manufacturer’s instructions. Cells were seeded at a density of 1.5 × 10^4^ cells/well with 250 µL DMEM glucose medium in XF24 cell culture plates and incubated for 24 h at 37 °C, 5% CO_2_. Then, cells were washed twice with 500 µL of a pre-warmed assay XF base medium (102353-100) supplemented with 10 mM D-glucose, 1 mM L-glutamine, and 1 mM sodium pyruvate and eventually 450 µL of an assay XF medium was added (final volume: 500 µL). Cells were incubated at 37 °C without CO_2_ for 1 h to allow pre-equilibrating with the assay medium.

Mitochondrial functionality was evaluated by the sequential injection of four compounds affecting bioenergetics. The final concentrations of the injected reagents were 1 µM oligomycin, 2 µM FCCP, and 1 and 2.5 µM rotenone/antimycin A. The best concentration of each inhibitor and uncoupler as well as the optimal cell seeding density were determined in preliminary analyses. A minimum of five wells per treatment were used in any given experiment. The studied parameters were the following: (1) Basal respiration: Oxygen consumption used to meet cellular ATP demand resulting from mitochondrial proton leak. It shows energetic demand of the cell under baseline conditions. (2) ATP production: The decrease in the oxygen consumption rate upon the injection of the ATP synthase inhibitor oligomycin represents the portion of basal respiration that was being used to drive ATP production. It shows ATP produced by the mitochondria that contributes to meeting the energetic needs of the cell. (3) Maximal respiration: The maximal oxygen consumption rate attained by adding the uncoupler FCCP. FCCP mimics a physiological “energy demand” by stimulating the mitochondrial respiratory chain to operate at maximum capacity to meet this metabolic challenge. It shows the maximum rate of respiration that the cell can achieve. (4) Spare respiratory capacity: This measurement indicates the capability of the cell to respond to an energetic demand as well as how closely the cell is to respire to its theoretical maximum.

### 2.14. Mitochondrial Complexes’ Activity

The activity of mitochondrial complex I and complex IV was performed according to the manufacturer’s instructions of the Complex I (ab109720) and Complex IV (ab109876) Enzyme Activity Dipstick Assay Kit, starting from cellular pellets. Signal intensity was acquired using the Chemidoc^TM^ MP Imaging System and analyzed using ImageLab^TM^ software version 6.1. (BioRad, Hercules, CA, USA).

### 2.15. SIRT3 Activity

Mitochondrial isolation was conducted using the Mitochondrial Isolation Kit for Cultured Cells (ab110170) (Abcam, Hercules, CA, USA). Then, SIRT3 activity was determined by the SIRT3 Activity Assay Kit (Fluorometric) (ab156067) in the mitochondrial fraction. Fluorescence was measured using a POLARstar Omega plate reader (BMG Labtech, Offenburg, Germany).

### 2.16. NAD^+^/NADH Levels

NAD^+^/NADH levels in cellular pellets were assessed by the NAD^+^/NADH Colorimetric Assay Kit (ab65348) protocol. The color intensity was measured using a POLARstar Omega plate reader.

### 2.17. Cell Transfection with Human LIPT1 Plasmid

The FLAG-tagged human LIPT1 plasmid (BC007001) was purchased from Sino Biological Inc. (Eschborn, Germany). An anti-DYKDDDDK tag antibody (A00187) was purchased from GenScripts (Piscataway, NJ, USA). Plasmid transfection was performed following the manufacturer’s instructions. Lipofectamine^®^ 2000 was purchased from ThermoFisher Scientific (Waltham, MA, USA).

### 2.18. Measurement of Cell Membrane and Mitochondrial Membrane Lipid Peroxidation

Lipid peroxidation was evaluated using 4,4-difluoro-5-(4-phenyl-1,3-butadienyl)-4-bora-3a,4a-diaza-s-indacene-3-undecanoic acid (BODIPY^®^ 581/591 C11) (D3861, ThermoFisher Scientific), a lipophilic fluorescent dye [24,25]. Cells were incubated with 5 μM BODIPY^®^ 581/591 C11 for 30 min at 37 °C. Luperox^®^ at 500 μM for 15 min was used as a positive control of lipid peroxidation. Nuclei were stained with 1 μg/mL DAPI. Lipid peroxidation in fibroblasts was evaluated using a light and fluorescence Axio Vert A1 microscope (Zeiss, Oberkochen, Germany) with a 20x objective. Images were analyzed with Fiji-ImageJ software version 2.9.0..

Mitochondrial lipid peroxidation was evaluated using a [3-(4-phenoxyphenylpyrenylphosphino) propyl] triphenylphosphonium iodide fluorescent probe (MitoPeDPP^®^) developed by Shioji K., et al. [26]. Fibroblasts were treated with 300 nM MitoPeDPP^®^ and 100 nM MitoTracker™ Red CMXRos, an in vivo mitochondrial membrane potential-dependent probe. Nuclei were stained with 1 μg/mL DAPI. The positive control of peroxidation was induced using 500 μM Luperox^®^ for 15 min. Images were taken in vivo using the DeltaVision system with an Olympus IX-71 fluorescence microscope with a 60x oil objective and analyzed by Fiji-ImageJ software version 2.9.0.

### 2.19. Direct Reprogramming

Neurons were generated from mutant and control fibroblasts by direct neuronal reprogramming as previously described by Drouin-Ouellet et al. [27,28]. Controls and mutant *LIPT1* patient-derived fibroblasts were plated on μ-Slide 4-Well Ibidi plates (Ibidi) and cultured in a DMEM Glutamax medium (10566016, ThermoFisher Scientific, Waltham, MA, USA) with a 1% Pen-Strep solution and 10% FBS.

The day after, dermal fibroblasts were transduced with one-single lentiviral vector containing neural lineage-specific transcription factors (Achaete-Scute Family BHLH Transcription Factor 1 (ASCL1), and POU class 3 homeobox 2 (BRN2)) and two shRNA against the REST complex, which were generated as previously described with a non-regulated ubiquitous phosphoglycerate kinase (PGK) promoter [29]. The plasmid was a gift from Dr. Malin Parmar (Developmental and Regenerative Neurobiology, Lund University, Sweden). Transduction was performed at a multiplicity of infection (MOI) of 30. On the following day, the cell culture medium was switched to a fresh DMEM Glutamax medium and after 48 h to an early neuronal differentiation medium (NDiff227) (Y40002, Takara-Clontech, Kusatsu, Japan) supplemented with neural growth factors and small molecules at the following concentrations: LM-22A4 (2 μM), GDNF (2 ng/mL), NT3 (10 ng/mL), dibutyryl cyclic AMP (db-cAMP, 0.5 mM), CHIR99021 (2 μM), SB-431542 (10 μM), noggin (50 ng/mL), LDN-193189 (0.5 M), and valproic acid (VPA, 1 mM). Half of the neuronal differentiation medium was refreshed every 2–3 days. Eighteen days post-infection, the medium was replaced by a late neuronal differentiation medium supplemented with only growth factors until the end of the cellular conversion. At day 21, cells were treated with CocT and the medium was changed every 2–3 days for 10 more days. Neuronal cells were identified by the expression of Tau protein. Nuclei were stained with 1 μg/mL DAPI. DAPI^+^/Tau^+^ cells were considered induced neurons (iNs). Conversion efficiency was calculated as the number of Tau^+^ cells over the total number of fibroblasts seeded for conversion. Neuronal purity was calculated as the number of Tau^+^ cells over the total cells in the plate after reprogramming.

### 2.20. Statistical Analysis

We used non-parametric statistics, where there were few events (*n <* 30), that do not have any distributional assumption, given the low reliability of normality testing for small sample sizes used in this work. In these cases, non-parametric methods such as Mann–Whitney were utilized in comparisons between two groups, while multiple groups were compared using a Kruskal–Wallis test. When the number of events was greater (*n >* 30), parametric methods were performed, specifically one-way ANOVA, comparing statistical differences between more than two groups. All results are expressed as the mean ± SD of 3 independent experiments and a *p*-value < 0.05 was considered as statistically significant. Statistical analyses were made with GraphPad Prism software version 9.4.1 (GraphPad Software, San Diego, CA, USA).

## 3. Results

### 3.1. LIPT1 Mutation Causes Deficiency of Lipoylation of PDH and KGDH E2 Subunits, Impaired 2-Ketoacid Dehydrogenase Enzymes’ Activities, and Iron Accumulation in Mutant Fibroblasts

**Figure 1:**
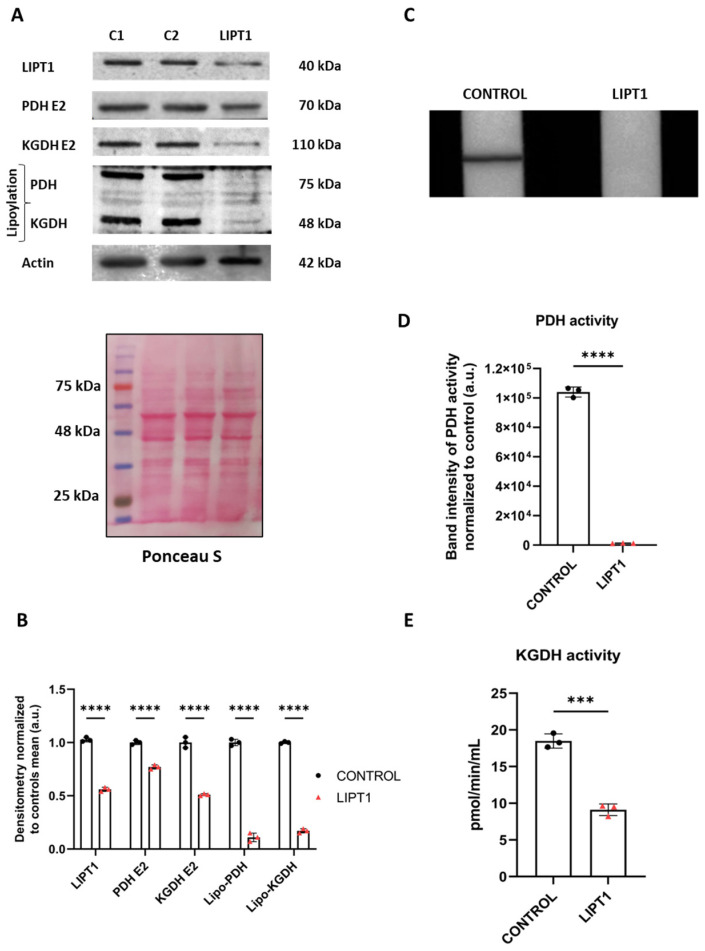
The characterization of the physiopathology of mutant *LIPT1* fibroblasts. (**A**). C1 and C2: control cells. The Western blot analysis of the mutated protein LIPT1, E2 subunits of multienzyme complexes PDH and KGDH, and their lipoylated form. Actin expression and Ponceau S staining were used to demonstrate equal protein loading. (**B**). Band densitometry of Western blot data referred to actin and normalized to the mean of controls. (**C**). PDH complex activity was measured by PDH Enzyme Activity Dipstick Assay Kit. (**D**). Band intensity of PDH complex activity was obtained by ImageLab software. (**E**). KGDH activity was measured by α-Ketoglutarate Dehydrogenase Activity Assay Kit. Data represent the mean ± SD of 3 independent experiments. *** *p* < 0.001 and **** *p* < 0.0001 between the control and patient’s fibroblasts. a.u.: arbitrary units.

First, we examined the physiopathological alterations of *LIPT1* mutation on patient-derived fibroblasts. The Western blot assay was performed to verify the expression levels of affected proteins including the mutated protein LIPT1 itself, mitochondrial lipoylated proteins, and the E2 subunits of multienzyme complexes PDH and KGDH (Figure 1A,B). Although LIPT1 and PDH E2 and KGDH E2 subunits’ expression levels were only mildly decreased, PDH and KGDH lipoylation was almost absent in the patient’s fibroblasts in comparison to control cells (Figure 1A,B).

Next, PDH activity was determined by a dipstick assay. As expected, no PDH activity was observed in mutant cells in comparison to control fibroblasts (Figure 1C,D). We also tested KGDH complex activity by a colorimetric assay. Results showed a significant reduction in KGDH activity in mutant cells in comparison to control cells (Figure 1E).

**Figure 2:**
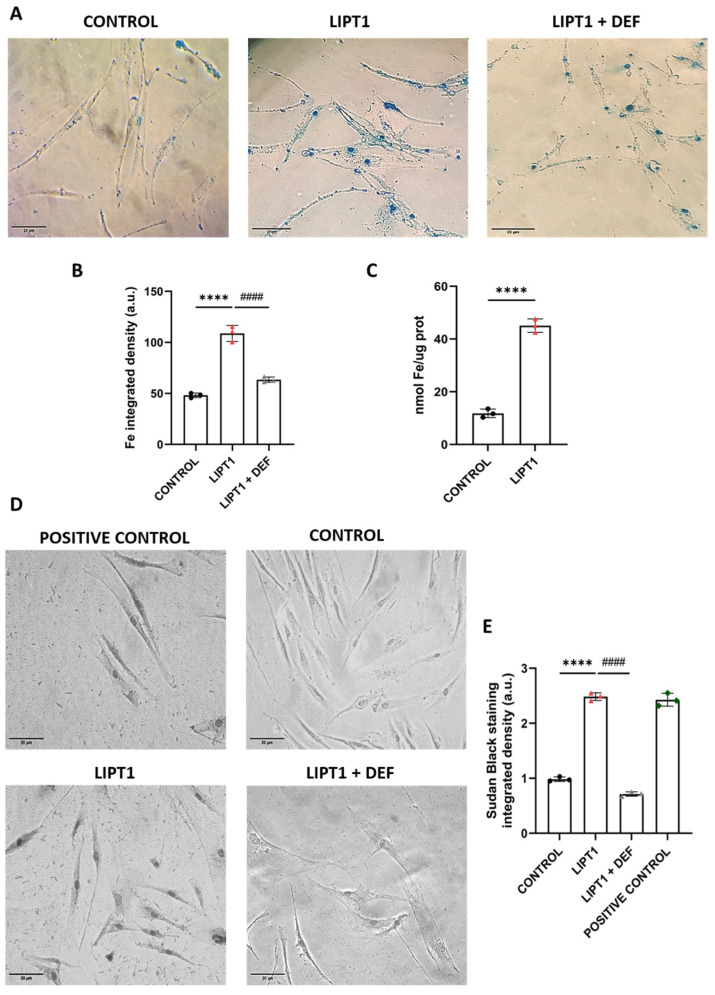
The analysis of iron accumulation in mutant *LIPT1* fibroblasts. (**A**). The control and patient’s fibroblasts (LIPT1) were stained with Prussian Blue staining. Mutant cells were treated with 100 µM Deferiprone (DEF). Images were acquired by a Zeiss Axio Vert A1 microscope. Scale bar: 20 µm. (**B**). The quantification of Prussian Blue staining-integrated density. Images were analyzed by the ImageJ software (at least 30 images were analyzed per each condition and experiment). (**C**). The quantification of iron content by ICP-MS. (**D**). Lipofuscin accumulation was assessed by Sudan Black staining. Mutant cells were treated with 100 µM DEF. Images were acquired by a Zeiss Axio Vert A1 microscope. A PKAN (pantothenate kinase-associated neurodegeneration) cell line was used as a positive control of lipofuscin accumulation. Scale bar: 20 µm. (**E**). The quantification of Sudan Black staining-integrated density (at least 30 images were analyzed per each condition and experiment). Data represent the mean ± SD of 3 independent experiments. **** *p* < 0.0001 between the control and patient’s fibroblasts. #### *p* < 0.0001 between mutant fibroblasts untreated and treated with Deferiprone. a.u.: arbitrary units.

On the other hand, as previous reports have highlighted the connections of LIPT1 deficiency and iron–sulfur protein biosynthesis, which are involved in the metabolism of iron in mitochondria [30] and LA metabolism [31], we assessed intracellular iron accumulation by Prussian Blue staining. Mutant *LIPT1* cells showed marked iron accumulation compared to control cells (Figure 2A,B). The patient’s fibroblasts were treated for 24 h with 100 μM Deferiprone, an iron-chelating drug, to confirm the specificity of the Prussian Blue staining for iron. Furthermore, iron overload in mutant *LIPT1* fibroblasts was confirmed by ICP-MS (Figure 2C). As iron can be accumulated in lipofuscin granules, the accumulation of this pigment was analyzed by Sudan Black staining. Mutant cells were treated with 100 μM Deferiprone for 24 h to confirm that lipofuscin accumulation is dependent on iron. The patient’s fibroblasts showed a significant increase in Sudan Black staining in comparison to control cells, indicating lipofuscin accumulation (Figure 2D,E).

### 3.2. Pharmacological Screening in Galactose Medium

Next, as LIPT1 is an important enzyme involved in the correct functioning of mitochondria [32] and previous works have shown that mutant *LIPT1* fibroblasts were unable to grow in a low-FBS galactose medium [14], we performed a pharmacological screening testing several compounds. Indeed, cultured mutant fibroblasts in the galactose medium lost their elongated shape, detached from the flask surface, and eventually died. This latter finding provides an excellent screening tool for the identification of pharmacological compounds capable of increasing cell viability and therefore with potential therapeutic interest.

Control and mutant cells were cultured for 3 days on the DMEM glucose medium untreated and treated with several antioxidants and mitochondrial cofactors to identify corrective supplements. Then, the medium was replaced by the nutritional stress medium with galactose and treatments were refreshed. The tested compounds were (1) biotin, a coenzyme necessary for the decarboxylation of enzymes associated with gluconeogenesis and fatty acid oxidation (FAO) [33]; (2) sodium pantothenate, required for the biosynthesis of coenzyme A, which is essential for KGDH and PDH complexes’ activity as well as FAO and many metabolic pathways [34]; (3) nicotinamide, an nicotinamide adenine dinucleotide (NAD^+^)precursor that also acts as a cofactor of sirtuins’ protein family [35]; (4) vitamin E, a membrane antioxidant and redox modulator [36]; (5) thiamine, an essential cofactor for the oxidative decarboxylation of multienzyme BCKDH complexes of the Krebs cycle [37]; and (6) α-LA, which, although it cannot be used directly for the lipoylation of mitochondrial enzymes, acts as a potent antioxidant [38]. In addition, we tested the combination of all these compounds (CocT): 5 μM biotin, 10 μM nicotinamide, 10 μM α-LA, 10 μM vitamin E, 10 μM thiamine, and 4 μM sodium pantothenate.

**Figure 3:**
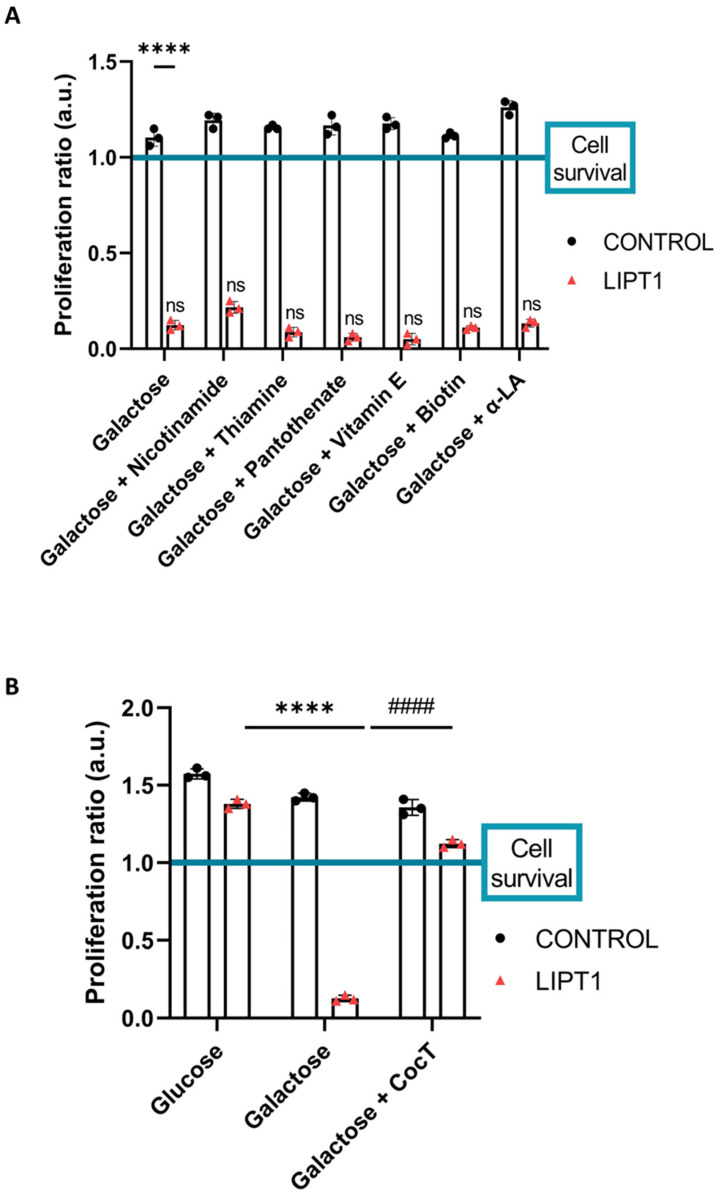
The quantification of the proliferation ratio in the galactose medium. Control and mutant cells (LIPT1) were seeded in the glucose medium and treated with the active compounds individually (**A**) and in a combination cocktail (**B**) for seven days. Then, the glucose medium was changed to the galactose medium, the treatment was renewed, and images were taken in that moment (T0) and 72 h later (T72) by BioTek Cytation 1 Cell Imaging Multi-Mode Reader. The proliferation ratio was calculated as the number of cells in T72 divided by the number of cells in T0, in both control and mutant cells (values > 1: cell proliferation; values = 1: number of cells unchanged; values < 1: cell death). Representative images are included in Supplementary Materials (Supplementary Figures S1 and S2). Data represent the mean ± SD of 3 independent experiments. **** *p* < 0.0001 between control and mutant fibroblasts in the galactose medium (**A**). **** *p* < 0.0001 between mutant fibroblasts in the glucose and in the galactose medium (**B**). #### *p* < 0.0001 between untreated and CocT-treated mutant *LIPT1* cells in the galactose medium. a.u.: arbitrary units. ns: not significant.

Control cells experienced no changes in the growth rate in the glucose medium or after switching to the galactose medium, as we expected. On the contrary, mutant *LIPT1* cells did not survive in the restrictive growth medium after 72 h. Then, we evaluated the effect of single supplements on cell survival. None of them were able to avoid cell death (Figure 3A and Supplementary Figure S1). Surprisingly, mutant *LIPT1* cells treated with a cocktail containing the combination of all the compounds (CocT) survived in the galactose medium (Figure 3B and Supplementary Figure S2). Moreover, different combinations of the compounds were tested, but none of them could avoid cell death in the galactose medium (Supplementary Figure S3).

In addition, control cells were treated with CPI-613 at 100 μM, a PDH inhibitor, to partially mimic the effects of *LIPT1* mutations (Supplementary Figure S4). In the glucose medium, almost no differences were seen in control versus treated cells. However, control cells treated with CPI-613 did not survive in the galactose medium. Interestingly, the effect of the inhibitor was partially reverted by the addition of CocT.

### 3.3. The Supplementation with CocT Increases LIPT1 Transcript Levels and Corrects Mutant Fibroblasts’ Pathophysiology

**Figure 4:**
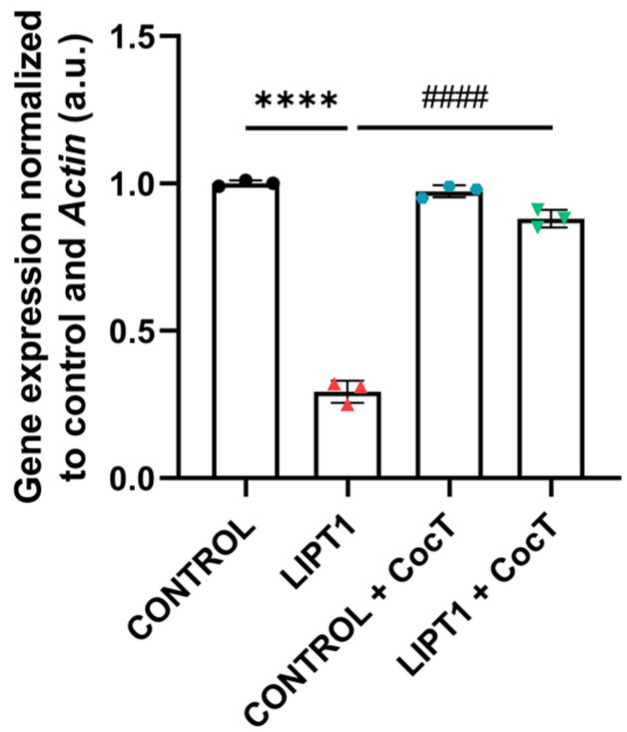
*LIPT1* transcript levels in mutant (LIPT1) and control fibroblasts with and without treatment. Cells were treated with CocT for seven days. Data represent the mean ± SD of 3 independent experiments. **** *p* < 0.0001 between control and mutant *LIPT1* fibroblasts. #### *p* < 0.0001 between untreated and treated mutant fibroblasts. a.u.: arbitrary units.

The pharmacological screening in the galactose medium identified a positive cocktail based on the combination of different well-known antioxidants and mitochondrial cofactors. The increased survival ratio in mutant *LIPT1* treated fibroblasts is likely due to a correction of metabolic alterations in mutant cells. For this reason, pathophysiological characterization assays were performed in untreated and treated control and mutant cells. In the first instance, we analyzed the transcript levels of *LIPT1* in both control and mutant fibroblasts, untreated and treated with CocT. We performed a qPCR assay and we observed that *LIPT1* transcript levels were downregulated in the patient’s cells in comparison to control cells. Interestingly, CocT supplementation induced a marked increase in *LIPT1* transcript levels (Figure 4).

**Figure 5:**
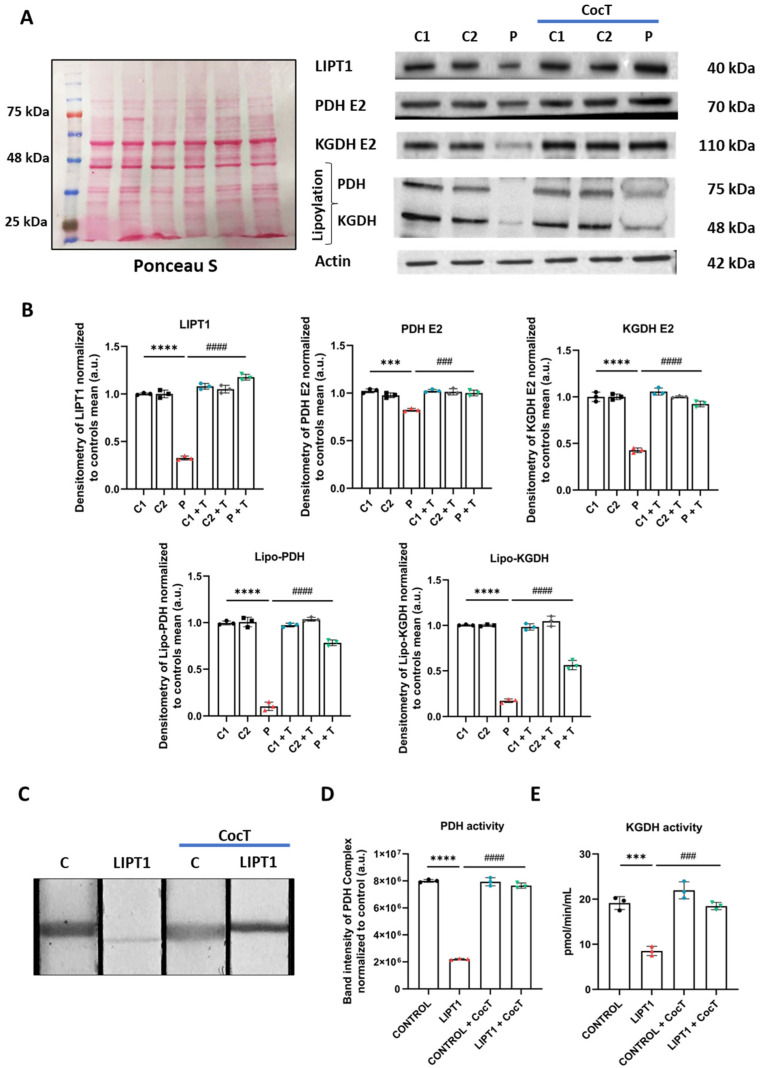
Expression levels of LIPT1, PDH E2, KGDH E2, and their lipoylated forms in control (C1, C2, and C) and mutant (LIPT1) fibroblasts before and after the supplementation with CocT. Cells were treated with CocT for seven days. (**A**). The Western blot analysis of the mutated protein LIPT1, E2 subunits of complexes PDH and KGDH, and their lipoylated forms. Actin expression and Ponceau S staining were used to demonstrate equal protein loading. (**B**). Band densitometry of Western blot data referred to actin and was normalized to the mean of controls. (**C**). PDH complex activity was measured by PDH Enzyme Activity Dipstick Assay Kit. (**D**). Band intensity of PDH complex activity was obtained by ImageLab software. (**E**). KGDH activity was measured by α-Ketoglutarate Dehydrogenase Activity Assay Kit. Data represent the mean ± SD of 3 independent experiments. *** *p* < 0.001 and **** *p* < 0.0001 between control and mutant LIPT1 fibroblasts. ### *p* < 0.001 and #### *p* < 0.0001 between untreated and treated mutant LIPT1 fibroblasts. a.u.: arbitrary units.

Then, we evaluated the effect of CocT treatment on the expression levels of affected proteins including the mutant protein LIPT1 itself, mitochondrial lipoylated proteins, and the E2 subunits of multienzyme complexes PDH and KGDH. Surprisingly, CocT supplementation induced a significant increase in all of them (Figure 5A,B). Consequently, CocT treatment also restored PDH and KGDH activities in mutant *LIPT1* cells (Figure 5C–E).

**Figure 6:**
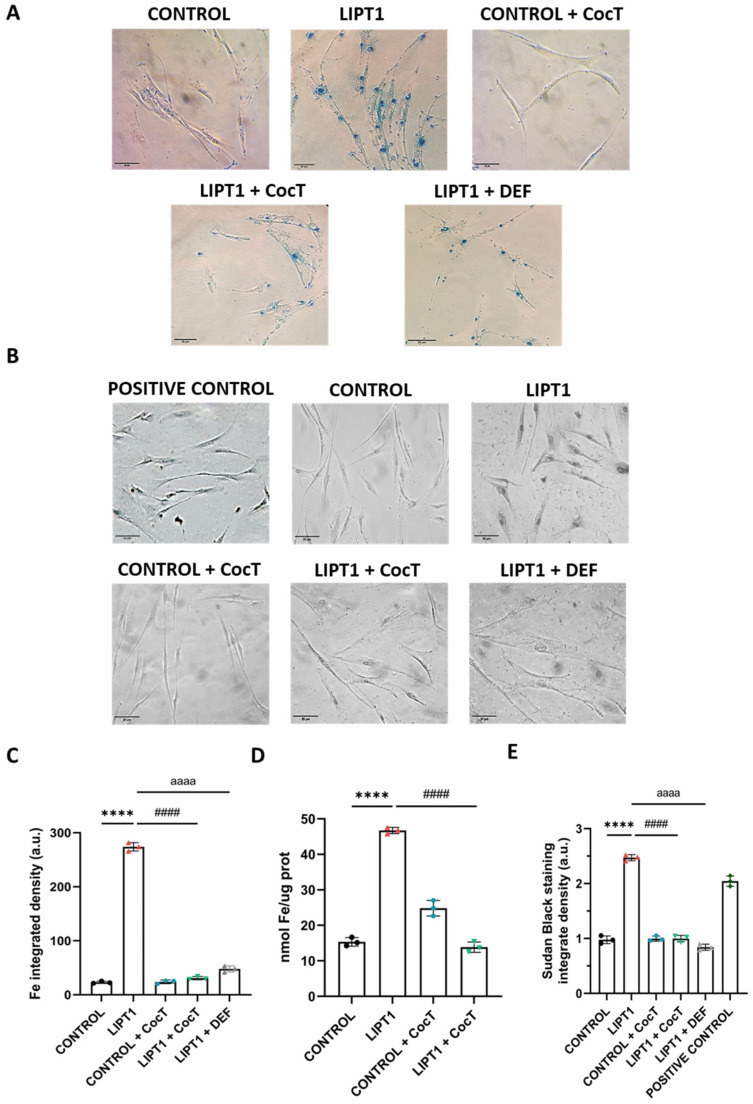
The effect of CocT on iron accumulation in mutant *LIPT1* fibroblasts. (**A**). Untreated and treated (for seven days) control and patient (LIPT1) fibroblasts were stained with Prussian Blue staining. (**B**). Lipofuscin accumulation was assessed by Sudan Black staining. A PKAN cell line was used as a positive control of lipofuscin accumulation [39]. Mutant cells were treated with 100 µM DEF. Representative images were acquired by a Zeiss Axio Vert A1 microscope (at least 30 images were analyzed per each condition and experiment). Scale bar: 20 µm. (**C**). The quantification of Prussian Blue staining-integrated density. Images were analyzed by ImageJ software (at least 30 images were analyzed per each condition and experiment). (**D**). The quantification of iron content by ICP-MS. (**E**). The quantification of Sudan Black staining-integrated density. Data represent the mean ± SD of 3 independent experiments. **** *p* < 0.0001 between control and mutant *LIPT1* fibroblasts. #### *p* < 0.0001 between untreated and treated mutant *LIPT1* fibroblasts. aaaa *p* < 0.0001 between untreated and treated mutant *LIPT1* fibroblasts with Deferiprone. a.u.: arbitrary units.

Next, we examined the effect of CocT supplementation on iron overload in mutant cells. Interestingly, iron accumulation, determined by Prussian Blue staining and mass spectrometry, was notably reduced in mutant cells after CocT treatment (Figure 6A,C,D). In the same way, lipofuscin accumulation, addressed by Sudan Black staining, was significantly decreased by CocT supplementation (Figure 6B,E). Additionally, mutant cells were treated with 100 μM Deferiprone to confirm the specificity of Prussian Blue staining for iron and the dependence of Sudan Black staining on the presence of this trace element (Figure 6).

**Figure 7:**
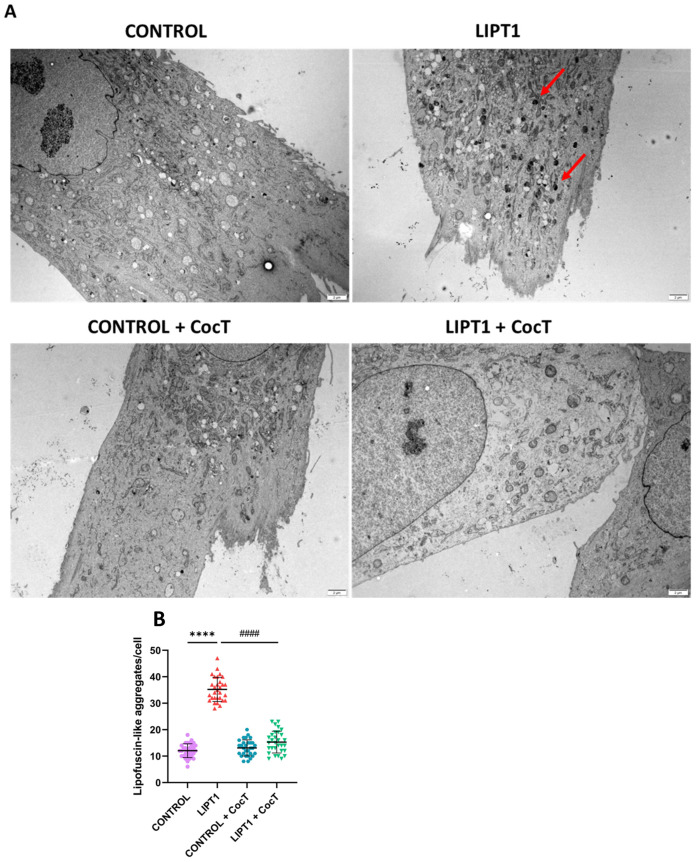
Electron microscopy images of the control and patient’s (LIPT1) fibroblasts, both untreated and treated with CocT. Cells were treated with CocT for seven days. (**A**). Representative electron microscopy images. Scale bar: 2 µm. Red arrows: lipofuscin-like granules. (**B**). The quantification of lipofuscin-like aggregates per cell (at least 30 images were analyzed per each condition and experiment). Data represent the mean ± SD of 3 independent experiments. **** *p* < 0.0001 between control and mutant *LIPT1* fibroblasts. #### *p* < 0.0001 between untreated and treated mutant *LIPT1* fibroblasts. Magnified images are shown in Supplementary Figure S5.

To confirm the presence of lipofuscin in *LIPT1* fibroblasts and the positive effect of CocT supplementation, an electron microscopy analysis was performed in control and mutant cells. We observed an accumulation of intracellular lipofuscin-like granules in mutant *LIPT1* cells in comparison to control fibroblasts, which was significantly reduced after CocT supplementation (Figure 7, Supplementary Figure S5).

**Figure 8:**
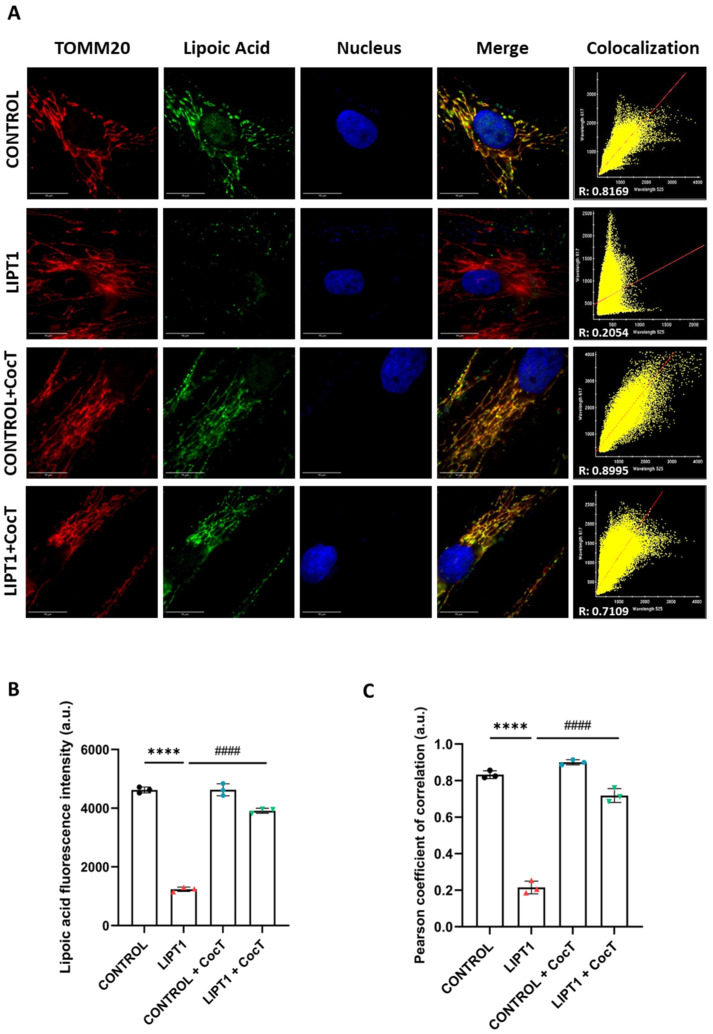
The effect of CocT on protein lipoylation. The immunofluorescence assay was performed in both untreated and treated control and mutant (LIPT1) fibroblasts. Cells were treated with CocT for seven days. (**A**). Cells were fixed and immunostained with the anti-LA antibody. TOMM20 was used as a mitochondrial marker and nuclei were visualized with DAPI staining. Scale bar: 15 µm. (**B**). The quantification of fluorescence intensity of the lipoic acid antibody. Images were analyzed by ImageJ software (at least 30 images were taken and analyzed from each condition and experiment). (**C**). The colocalization between lipoic acid and TOMM20 signals was analyzed by the Pearson correlation coefficient. The Pearson correlation coefficient was calculated by the DeltaVision system. Data represent the mean ± SD of 3 independent experiments. **** *p* < 0.0001 between control and mutant *LIPT1* fibroblasts. #### *p* < 0.0001 between untreated and treated mutant *LIPT1* fibroblasts. a.u.: arbitrary units.

Then, an immunofluorescence assay was performed to confirm the effect of CocT on protein lipoylation in mutant *LIPT1* cells. As expected, we observed a notable reduction in lipoic acid fluorescence intensity in mutant cells in comparison to control cells (Figure 8A). Interestingly, the supplementation of CocT induced a significant increase in lipoic acid fluorescence intensity in mutant fibroblasts. Additionally, as we used the Translocase of Outer Mitochondrial Membrane (TOMM20) as a mitochondrial marker, we confirmed the localization of protein lipoylation in mitochondria (Figure 8C).

To confirm the role of LIPT1 in iron metabolism and protein lipoylation, we next performed cDNA complementation assays, in which we introduced FLAG-tagged human *LIPT1* cDNA into control and mutant fibroblasts. The immunofluorescence analysis using an anti-lipoic and anti-FLAG antibody showed that mutant cells expressed low levels of protein lipoylation and no FLAG signal was detected. However, patient cells expressing recombinant *LIPT1* (r*LIPT1*) had higher protein lipoylation and FLAG signals (Supplementary Figure S6). Both signals showed high colocalization with a Pearson’s correlation coefficient > 0.90. We then analyzed iron overload by Prussian Blue staining. The expression of recombinant *LIPT1* significantly eliminated iron accumulation in mutant cells (Supplementary Figure S7). Thus, these data demonstrate a direct link between the expression of *LIPT1* and protein lipoylation and iron overload.

### 3.4. CocT Supplementation Increases Mitochondrial Proteins’ Expression Levels and Improves Mitochondrial Bioenergetics of Mutant LIPT1 Cells

**Figure 9:**
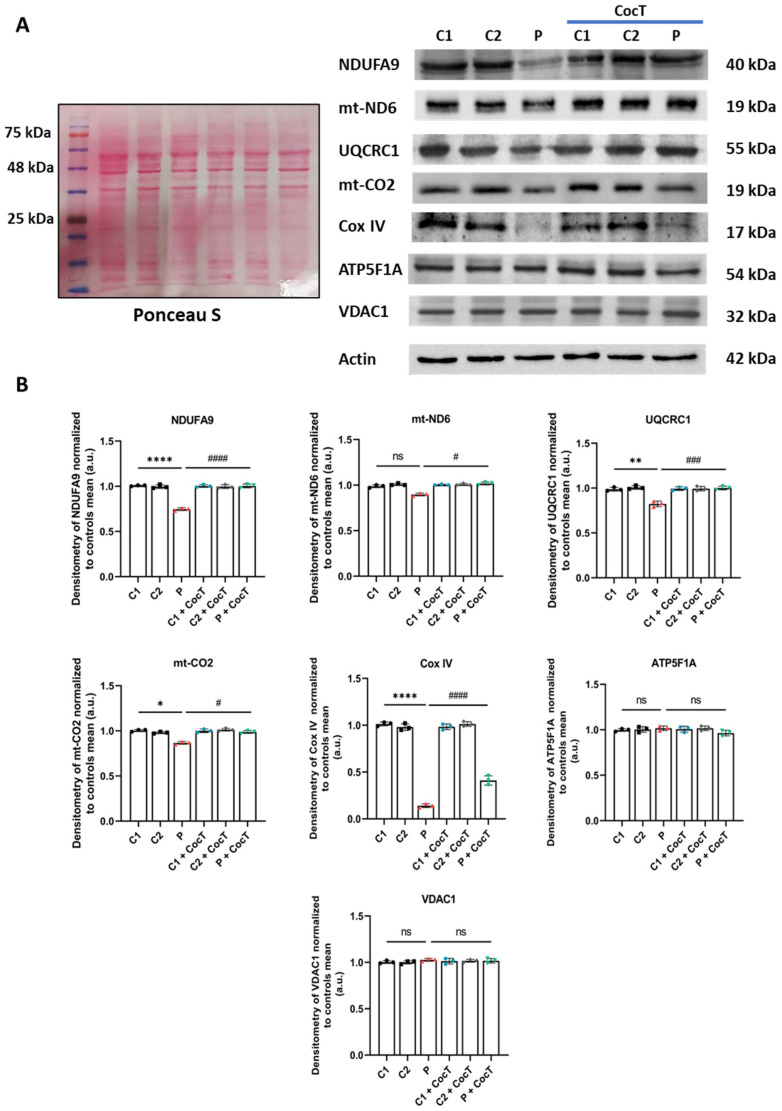
The effect of CocT on the expression levels of subunits of the mitochondrial electron transport chain (mtETC) complexes in control (C1 and C2) and mutant (LIPT1) cells. Cells were treated with CocT for seven days. (**A**). The Western blot analysis of proteins of complex I (NDUFA9 and mt-ND6), complex III (UQCRC1), complex IV (mt-CO2 and COX IV), and complex V (ATP5F1A). Actin expression and Ponceau S staining were used to demonstrate equal protein loading. VDAC1 was used as a mitochondrial mass marker. (**B**). Band densitometry of Western blot data referred to actin and was normalized to the mean of controls. Data represent the mean ± SD of 3 independent experiments. * *p* < 0.05, ** *p* < 0.01, and **** *p* < 0.0001 between control and mutant *LIPT1* fibroblasts. # *p* < 0.05, ### *p* < 0.001, and #### *p* < 0.0001 between untreated and treated mutant *LIPT1* fibroblasts. a.u.: arbitrary units. ns: not significant.

Due to the essential role of LIPT1 and protein lipoylation in mitochondrial function, we then examined the expression levels of mitochondrial proteins and mitochondrial bioenergetics in control and mutant cells with and without the supplementation of CocT. First, to confirm the effect of CocT supplementation on mitochondrial dysfunction, several subunits of mitochondrial complexes, codified by the nuclear DNA (nDNA) or mitochondrial DNA (mtDNA), were analyzed by a Western blot (Figure 9). Complex I subunit NDUFA9, complex III subunit UQCRC1, and complex IV subunits (COX IV and mt-CO2) protein expression levels were markedly reduced in mutant cells. However, the protein expression levels of mt-ND6 (complex I), ATP5F1A (complex V), and VDAC1 (a mitochondrial mass marker) remain without significant changes in control and mutant *LIPT1* cells. Interestingly, the expression levels of mitochondrial proteins that were downregulated in mutant fibroblasts were significantly restored by CocT supplementation.

**Figure 10:**
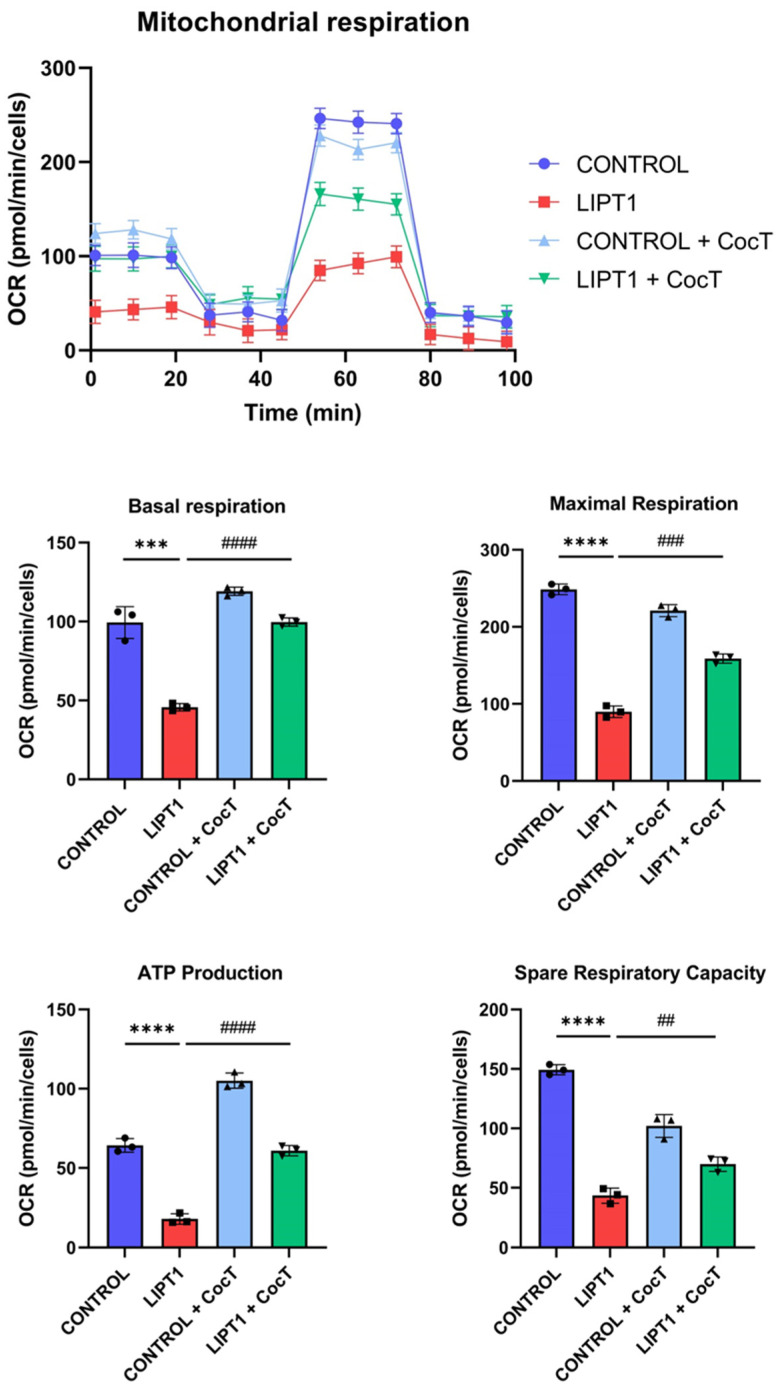
The effect of CocT on mitostress bioenergetic assay control and mutant (LIPT1) fibroblasts. Cells were treated with CocT for seven days. The mitochondrial respiration profile was measured using a Seahorse XFe24 analyzer. Data represent the mean ± SD of 3 independent experiments. *** *p* < 0.001, and **** *p* < 0.0001 between control and mutant *LIPT1* fibroblasts. ## *p* < 0.01, ### *p* < 0.001 and #### *p* < 0.0001 between untreated and treated mutant *LIPT1* fibroblasts. OCR: Oxygen Consumption Rate.

Subsequently, to assess the bioenergetic profile of mutant fibroblasts and evaluate the effect of CocT on bioenergetic parameters, a Mitostress SeaHorse assay was performed. Mutant *LIPT1* cells showed a marked reduction in all analyzed bioenergetic parameters (basal respiration, maximal respiration, ATP production, and spare respiratory capacity) compared to control cells. Curiously, CocT supplementation induced a significant improvement in mutant *LIPT1* cells (Figure 10).

**Figure 11:**
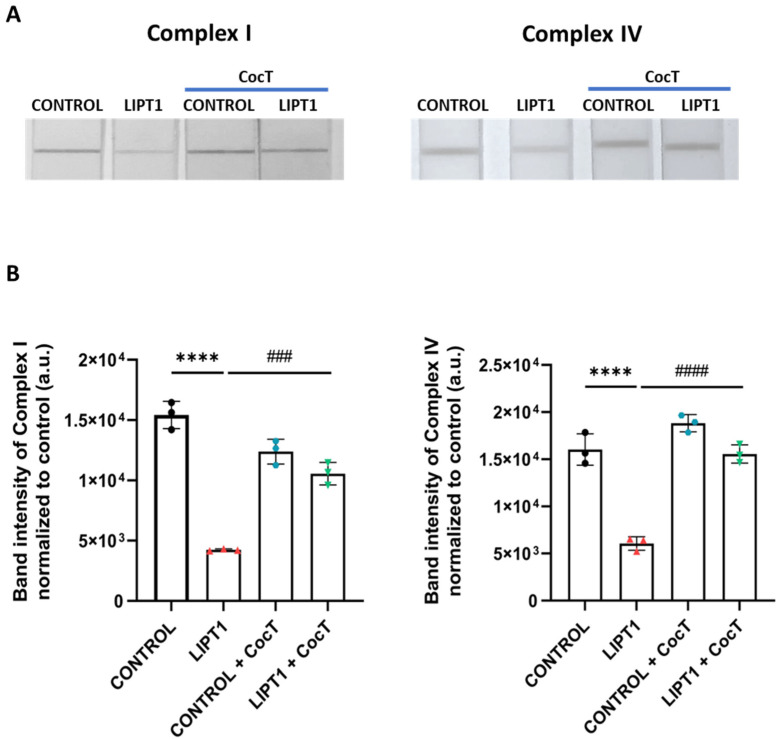
The effect of CocT on complex I and complex IV activities in control and mutant (LIPT1) fibroblasts. Cells were treated with CocT for seven days. (**A**). Complex I activity was measured using Complex I Enzyme Activity Dipstick Assay Kit. Complex IV activity was measured using Complex IV Enzyme Activity Dipstick Assay Kit. (**B**). Band intensity was obtained using ImageLab software. Data represent the mean ± SD of 3 independent experiments. **** *p* < 0.0001 between control and mutant *LIPT1* fibroblasts. ### *p* < 0.001 and #### *p* < 0.0001 between untreated and treated mutant *LIPT1* fibroblasts. a.u.: arbitrary units.

To corroborate mitochondrial function improvement, complex I and complex IV activities were measured by a dipstick assay. Both complexes’ activities were reduced in mutant fibroblasts in comparison to control cells. Interestingly, CocT treatment restored both complex I and complex IV activities in the patient’s fibroblasts (Figure 11).

As mitochondrial dysfunction is associated with Reactive Oxygen Species (ROS) overproduction, which induces oxidative cell membrane damage, the lipid peroxidation of mitochondrial and cell membranes was examined. High levels of lipid peroxidation in mitochondrial and cell membranes were observed in mutant cells in comparison to control fibroblasts. As expected, peroxidation levels in cell and mitochondrial membranes were significantly reduced after CocT treatment (Supplementary Figures S8 and S9).

### 3.5. Supplementation with CocT Activates the Mitochondrial Unfolded Protein Response (mtUPR) and Mitochondrial Biogenesis

**Figure 12:**
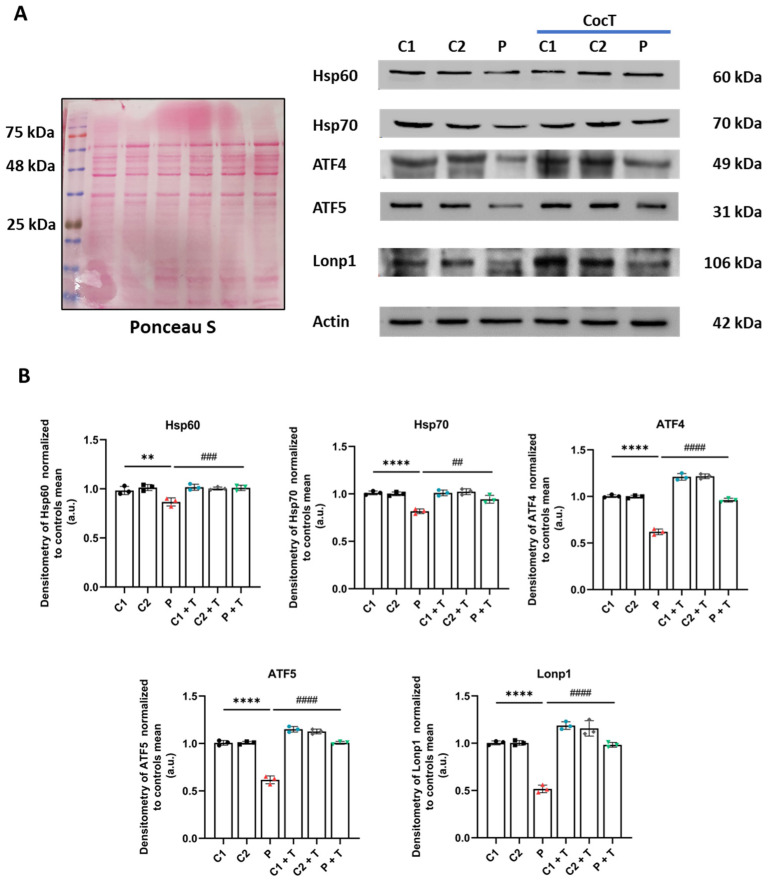
The effect of CocT on the expression levels of proteins of the transcriptional canonical mtUPR axis in control (C1 and C2) and mutant (LIPT1) cells. Cells were treated with CocT for seven days. (**A**). The Western blot analysis of transcriptional canonical mtUPR proteins. Actin expression and Ponceau S staining were used to demonstrate equal protein loading. (**B**). Band densitometry of Western blot data referred to actin and was normalized to the mean of controls. Data represent the mean ± SD of 3 independent experiments. ** *p* < 0.01 and **** *p* < 0.0001 between control and mutant *LIPT1* fibroblasts. ## *p* < 0.01, ### *p* < 0.001, and #### *p* < 0.0001 between untreated and treated mutant *LIPT1* fibroblasts. a.u.: arbitrary units.

Next, we evaluated whether the beneficial effect of CocT was mediated by the activation of mtUPR, a well-known mitochondrial compensatory pathway. Although mtUPR was first described as a transcriptional response resulting in an increase in chaperones and proteases’ expression to protect cells from the accumulation of misfolded or unfolded proteins [40], it was recently described as more than one axis [41]. In this work, we focused on two mtUPR axes. The first one is known as the transcriptional canonical axis of mtUPR, which induces the expression of mitochondrial chaperones and proteases. We observed a reduction in the expression levels of ATF4, ATF5, Hsp60, Hsp70, and Lonp1 in the patient’s cells in comparison to control fibroblasts, which were notably upregulated after CocT supplementation (Figure 12).

**Figure 13:**
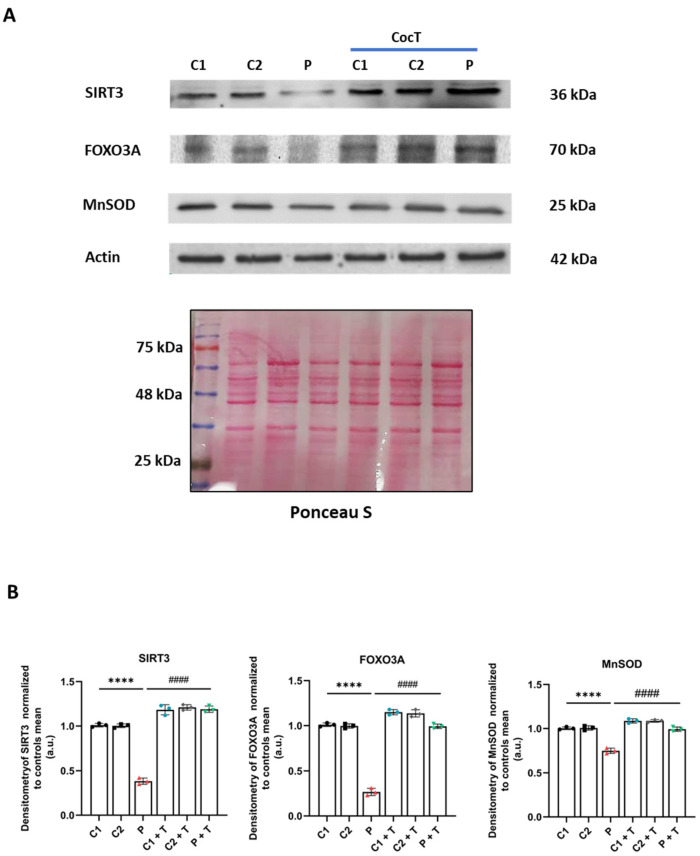
The effect of CocT on the expression levels of proteins of the SIRT3 mtUPR axis in control (C1 and C2) and mutant (LIPT1) cells. Cells were treated with CocT for seven days. (**A**). The Western blot analysis of SIRT3 mtUPR proteins. Actin expression and Ponceau staining were used to demonstrate equal protein loading. (**B**). Band densitometry of Western blot data referred to actin and was normalized to the mean of controls. Data represent the mean ± SD of 3 independent experiments. **** *p* < 0.0001 between control and mutant *LIPT1* fibroblasts. #### *p* < 0.0001 between untreated and treated mutant *LIPT1* fibroblasts. a.u.: arbitrary units.

Subsequently, we studied the second axis, known as SIRT3 mtUPR, and we examined the expression levels of SIRT3, whose activation promotes the expression of downstream antioxidant enzyme proteins such as MnSOD or catalase through the deacetylation and activation of the transcription factor FOXO3A [42]. We observed that the expression levels of SIRT3, FOXO3A, and MnSOD were downregulated in the patient’s fibroblasts in comparison to control cells, and they were significantly increased after CocT treatment (Figure 13).

**Figure 14:**
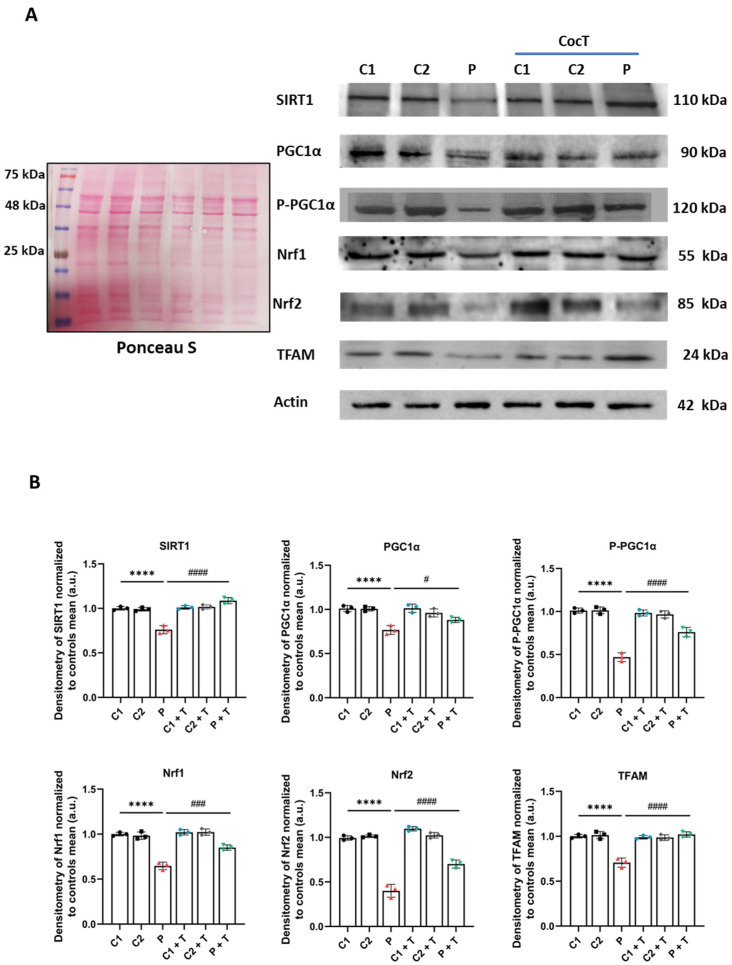
The effect of CocT on the expression levels of mitochondrial biogenesis proteins in control (C1 and C2) and mutant (LIPT1) cells. Cells were treated with CocT for seven days. (**A**). The Western blot analysis of mitochondrial biogenesis proteins. Actin expression and Ponceau S staining were used to demonstrate equal protein loading. (**B**). Band densitometry of Western blot data referred to actin and was normalized to the mean of controls. Data represent the mean ± SD of 3 independent experiments. **** *p* < 0.0001 between control and mutant *LIPT1* fibroblasts. # *p* < 0.05, ### *p* < 0.001, and #### *p* < 0.0001 between untreated and treated mutant *LIPT1* fibroblasts. a.u.: arbitrary units.

In addition, we assessed the expression levels of proteins involved in mitochondrial biogenesis. The expression levels of all the analyzed proteins, SIRT1, (P)PGC1α, Nrf1, Nrf2, and TFAM, were downregulated in mutant fibroblasts in comparison to control cells, and CocT supplementation was able to partially restore protein expression levels (Figure 14).

**Figure 15:**
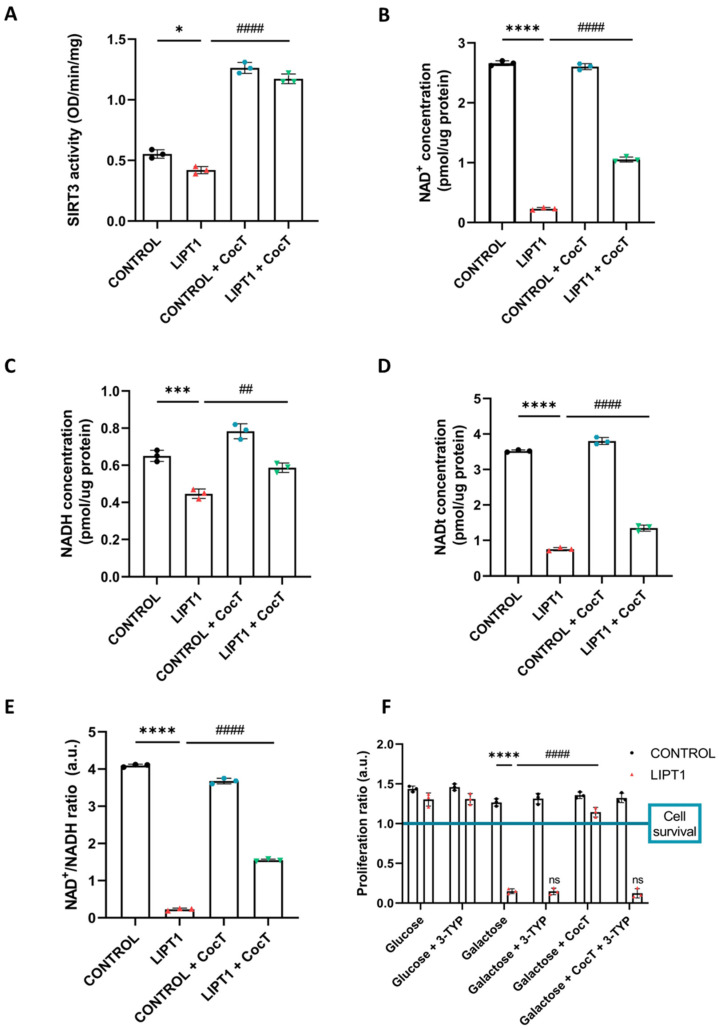
The effect of CocT on SIRT3 activity and NAD^+^, NADH, NADt, and NAD^+^/NADH ratio levels in control and mutant (LIPT1) fibroblasts. Cells were treated with CocT for seven days. (**A**). Mitochondrial SIRT3 activity was determined by SIRT3 Activity Assay Kit (Fluorometric) in mitochondrial fractions. (**B**). The effect of CocT on cellular NAD^+^ levels. (**C**). The effect of CocT on cellular NADH levels. (**D**). The effect of CocT on cellular NADt levels. (**E**). The effect of CocT on cellular NAD^+^/NADH ratio levels. (**F**). Consequences of SIRT3 inhibition in CocT positive effects. The quantification of the proliferation ratio of pharmacological screening in the galactose medium with 3-TYP, a SIRT3 inhibitor. Control and mutant (LIPT1) cells were seeded in the glucose medium and treated with CocT for seven days along with 50 nM 3-TYP (added for 72 h).Then, the glucose medium was changed to the galactose medium, treatment was refreshed, and photos were taken at that moment (T0) and 72 h later (T72) by BioTek Cytation 1 Cell Imaging Multi-Mode Reader. The proliferation ratio was calculated as the number of cells in T72 divided by the number of cells in T0, in both control and mutant cells (values > 1: cell proliferation; values = 1: number of cells unchanged; values < 1: cell death). Data represent the mean ± SD of 3 independent experiments. * *p* < 0.05, *** *p* < 0.001, and **** *p* < 0.0001 between control and mutant *LIPT1* fibroblasts. ## *p* < 0.01 and #### *p* < 0.0001 between untreated and treated mutant *LIPT1* fibroblasts. a.u.: arbitrary units.

To explore the molecular mechanism involved in the beneficial effect of CocT supplementation, we assessed its effect on SIRT3 activity. Due to it being the predominant sirtuin within mitochondria, we decided to isolate the mitochondrial fraction from cell extracts using a cell fractioning protocol. SIRT3 activity was determined in the mitochondrial fraction and results showed that SIRT3 activity was partially reduced in mutant cells in comparison to control fibroblasts. After CocT supplementation, SIRT3 activity was significantly increased, even in control cells, suggesting that the treatment was activating it (Figure 15A).

The purity of the mitochondrial fraction was confirmed by using protein markers of nuclear (phosphorylated H2A histone family member X (H2AX)), cytoplasmatic (tubulin), and mitochondrial (VDAC1) fractions (Supplementary Figure S10).

For deacetylation reactions, sirtuins need NAD^+^ as a cofactor to remove the acetyl group from their different substrates. Moreover, it is well known that an imbalance between NADH and NAD^+^ can promote an impairment in cell metabolism and limits the usage of NAD^+^ by sirtuins [43]. For this reason, NADH, NAD^+^, and NAD total (NADt) levels, as well as the NADH/NAD^+^ ratio, were determined, before and after the supplementation with CocT (Figure 15). NAD^+^ levels (Figure 15B), NADH levels (Figure 15C), NADt levels (Figure 15D), and the NAD^+^/NADH ratio (Figure 15E) were markedly downregulated in mutant fibroblasts in comparison to control cells. After CocT supplementation, mutant fibroblasts significantly recovered NAD^+^, NADt, and NADH levels as well as the NAD^+^/NADH ratio.

### 3.6. 3-TYP, a Specific SIRT3 Inhibitor, Blocks the Effect of CocT

Next, to confirm the effect of CocT on SIRT3 activation, we used 3-TYP, a selective inhibitor of SIRT3. Thus, we examined the effect of 3-TYP on the screening assay with the galactose medium. As we expected, 3-TYP had no negative consequences on control fibroblasts and neither the glucose nor galactose medium after 72h. Nevertheless, mutant fibroblasts did not survive in the presence of 3-TYP after 72 h in the galactose medium, even under CocT treatment (Figure 15F and Supplementary Figure S11). In addition, 3-TYP prevented the increase in protein lipoylation in mutant cells under CocT treatment (Supplementary Figure S12). These findings suggest that SIRT3 inhibition avoids the positive effect of CocT supplementation.

### 3.7. Induced Neurons

Patient-derived fibroblasts as cellular models provided useful information on the pathophysiology of this disease. However, the most affected cell types in most metabolic mitochondrial pathologies are those with high energy requirements, such as muscle cells and neurons [44,45]. Therefore, direct reprogramming of patient-derived fibroblasts into iNs is an extremely valuable tool to understand the pathogenesis of these disorders. For this reason, control and mutant fibroblasts were directly reprogrammed to iNs. Reprogrammed cells presented a typical neuron-like morphology and positive immunoreactivity against Tau, a microtubule-associated protein primarily found in neuronal axons of vertebrates’ brain. In contrast, unprogrammed cells did not show Tau staining.

Tau^+^ cells were used to calculate neuronal conversion efficiency (Tau^+^ cells over the total number of fibroblasts seeded for conversion), which was around 22% in control cells (20.3% ± 1.4%) and 20% (19.2% ± 2.2%) in mutant *LIPT1* cells. Neuronal purity (Tau^+^ cells over the total cells in the plate after reprogramming) was around 95% (93.6% ± 3.2%) in control cells and up to 86% (85.1% ± 1.6%) in mutant *LIPT1* cells (Supplementary Figure S13).

**Figure 16:**
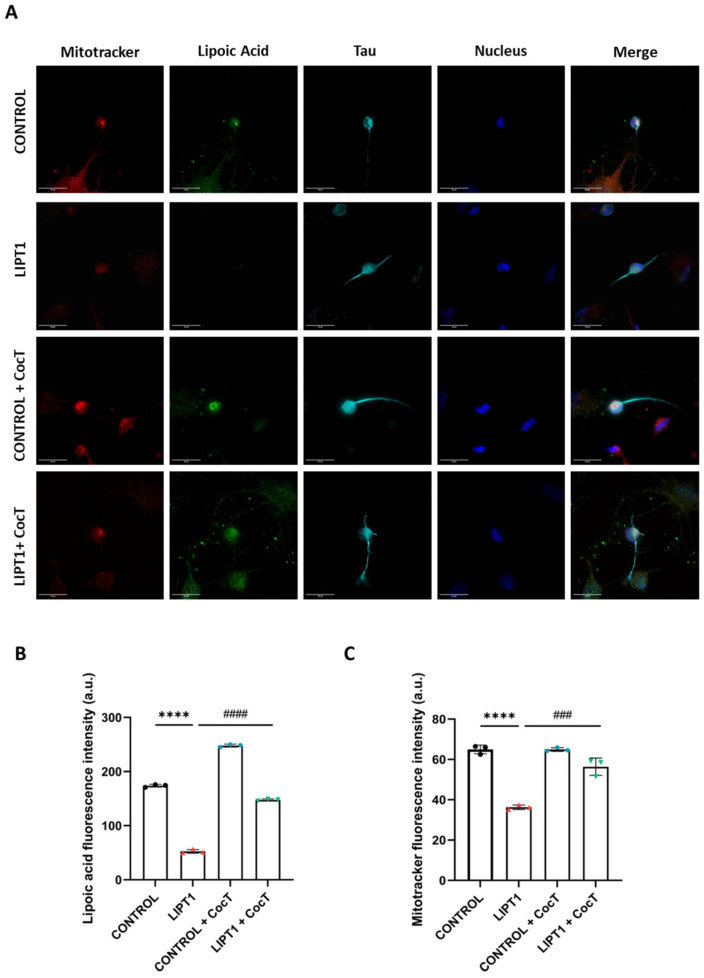
The effect of CocT supplementation on protein lipoylation in iNs. Control and mutant (LIPT1) cells, reprogrammed from fibroblasts to iNs, were treated with CocT for seven days. (**A**). iNs were fixed and immunostained with the anti-LA antibody. The mitochondrial network was assessed by MitoTracker^TM^ Red CMXRos staining. Tau was used as a neuronal marker. Hoescht was used to stain nuclei. Scale bar: 15 μm. (**B**). The quantification of fluorescence intensity of the lipoic acid antibody. (**C**). The quantification of fluorescence intensity of Mitotracker^TM^ Red CMXRos. Images were analyzed by Image J software (at least 30 images were analyzed per each condition and experiment). **** *p* < 0.0001 between control and mutant *LIPT1* iNs. ### *p* < 0.001 and #### *p* < 0.0001 between untreated and treated mutant *LIPT1* iNs. a.u.: arbitrary units.

Then, efficacy of CocT treatment was evaluated in control and mutant *LIPT1* iNs. Overall, the lipoylation of proteins was studied with an immunofluorescence assay. Additionally, the mitochondrial network was assessed by MitoTracker^TM^ Red CMXRos. In mutant *LIPT1* iNs, lipoylation was almost totally absent. CocT supplementation partially reverted the lipoylation levels on mutant *LIPT1* iNs as previously seen in fibroblasts (Figure 16).

**Figure 17:**
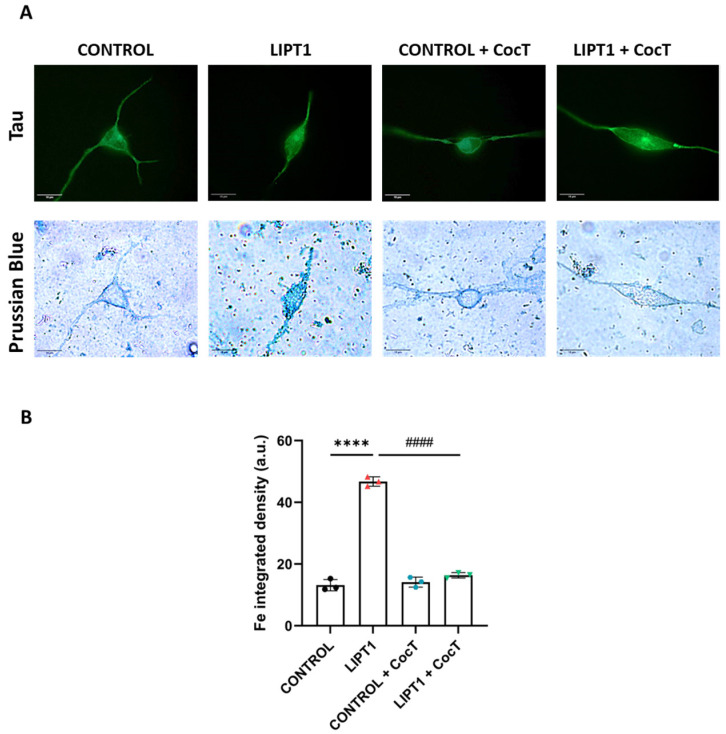
The effect of CocT on iron accumulation in iNs generated from control and patient-derived fibroblasts by direct reprogramming. Control and mutant (LIPT1) iNs were treated with CocT for seven days. (**A**). Representative images were acquired by a Zeiss Axio Vert A1 microscope. Tau was used as a neuronal marker. Scale bar: 15 μm. (**B**). Quantification of Prussian Blue staining images were obtained by Image J software (at least 30 images were analyzed per each experimental condition). **** *p* < 0.0001 between control and mutant *LIPT1* iNs. #### *p* < 0.0001 between untreated and treated mutant *LIPT1* iNs. a.u.: arbitrary units.

To continue to study the physiopathology on the iNs, we evaluated intracellular iron accumulation. As observed in fibroblasts, mutant iNs showed iron overload, and the supplementation of CocT significantly reduced iron accumulation to the levels of control iNs (Figure 17).

## 4. Discussion

In this article, we examined the pathophysiological alterations in cellular models derived from a mutant *LIPT1* patient. To address the pathological consequences of the mutation, we evaluated mitochondrial proteins’ expression levels and mitochondrial function. Mutant cells showed reduced expression levels of the LIPT1 enzyme and mitochondrial lipoylated proteins, associated with impaired mitochondrial function and iron accumulation as well as reduced mitochondrial membrane potential and increased oxidative stress and lipid peroxidation. Interestingly, the supplementation with α-LA, nicotinamide, sodium pantothenate, vitamin E, thiamine, and biotin in a cocktail (CocT) during seven days was able to correct the main physiopathological alterations. This cocktail enabled mutant *LIPT1* cells to survive in the nutrient stress medium and significantly corrected protein lipoylation, tricarboxylic acid (TCA) cycle enzymes’ activity, and consequently mitochondrial function.

α-LA is an essential cofactor for mitochondrial metabolism whose exogenous supplementation is not able to lipoylate mitochondrial proteins in humans [46]. Thus, α-LA must be synthesized de novo within mitochondria using intermediates from mitochondrial fatty acid synthesis, S-adenosylmethionine and iron–sulfur clusters [47]. Therefore, any mutation affecting the α-LA biosynthetic pathway is responsible for severe metabolic and mitochondrial alterations [13,48]. The LIPT1 enzyme is involved in the protein lipoylation of essential mitochondrial enzymes such as 2-ketoacid dehydrogenase complexes (mainly PDH and KGDH). Consequently, mutant *LIPT1* patient-derived fibroblasts showed a marked reduction in PDH and KGDH lipoylation (Figure 1A) as well as a pronounced decrease in PDH (Figure 1D) and KGDH (Figure 1E) activities. Both enzymes are essential for TCA cycle functioning and consequently for mitochondrial energy production.

Another pathological consequence of mitochondrial lipoylation deficiency in mutant cells was intracellular iron accumulation. It has been reported that mutations causing impairment of the PDH E2 subunit lead to PDH activity deficiency and cause a type of Leigh disease, in which neuroradiographic abnormalities indicating iron accumulation were observed, specifically in the globus pallidus [49,50,51]. The symptoms, signs, and magnetic resonance imaging (MRI) characteristics of PDH E2 deficiency can be similar to pantothenate kinase-associated neurodegeneration (PKAN), a subtype of neurodegeneration with brain iron accumulation (NBIA) disorders. Interestingly, mutant *LIPT1* fibroblasts showed intracellular iron accumulation (Figure 2A) to a similar extent to PKAN cellular models [52,53]. The clinical and neuroradiographic overlapping features of PKAN and PDH E2 deficiency as well as CoA synthase protein-associated neurodegeneration (CoPAN), and Mitochondrial Enoyl CoA Reductase protein-associated neurodegeneration (MePAN), suggest a common element in their pathogeneses [54].

Cellular or radiological evidence of iron accumulation has not been described in other mutations involved in lipoate synthesis such as LIPT2 and LIAS [4]. However, considering the intricate relationship among mtFASII, iron–sulfur cluster (ISC) biosynthesis, cellular iron homeostasis, and α-LA synthesis and mitochondrial protein lipoylation, deficiencies in proteins implicated in these processes can lead to iron accumulation [4,32,53,54,55,56,57].

Although the mechanism underlying intracellular iron accumulation in *LIPT1* mutations is unknown, it has been demonstrated that α-LA is implicated in iron metabolism [56] and mitochondrial iron–sulfur cluster biosynthesis [32]. Moreover, a Sudan Black staining assay was performed to see if iron overload leads to lipofuscin accumulation. Lipofuscin is an autofluorescent pigment that accumulates in cells through aging [58,59] and the accumulation of dysfunctional mitochondria might be responsible for lipofuscinogenesis [60,61]. Corroborating this hypothesis, mutant fibroblasts presented a significant increase in lipofuscin granules (Figure 2E and Figure 7).

Next, with the objective of identifying potential therapeutic approaches for this severe disease, we developed a cellular screening assay based on the capability of cells to survive in the galactose medium. Mutant *LIPT1* fibroblasts manifested a profound mitochondrial dysfunction and were unable to survive in the restrictive galactose medium. Next, several compounds identified in previous studies were evaluated [62,63], including α-LA supplementation (Figure 3A and Supplementary Figure S1). Nevertheless, none of them individually improved cell survival under stress conditions. The lack of an independent salvage pathway in humans, such as an exogenous α-LA integration to lipoylation, abrogates the use of α-LA supplementation as a direct therapeutic option in α-LA biosynthesis- and protein transfer-related mutations [6]. However, α-LA is a pleiotropic molecule with several functions in the organism and has been used as a therapeutic agent for cardiovascular diseases, hypertension, and diabetes [64,65]. In addition, α-LA treatment has been reported to provide neuroprotection against Parkinson’s disease [66], aging [67], and memory loss [68] because it can penetrate the blood–brain barrier, although the associated mechanisms remain unclear. Furthermore, α-LA is considered a chelator and therefore can reduce iron in cells and tissues [69,70]. Recently, our group has demonstrated the beneficial effect of α-LA supplementation on cellular models of PKAN [39].

Given that individually, the compounds had no positive effect, we then combined all of them in a cocktail (CocT), which contained 5 μM biotin, 10 μM nicotinamide, 10 μM α-LA, 10 μM vitamin E, 10 μM thiamine, and 4 μM sodium pantothenate. Surprisingly, mutant fibroblasts survived in the stress galactose medium after supplementation with CocT (Figure 3B, Supplementary Figures S2 and S3).

Our next step was to assess whether the survival of mutant cells in the stress medium was associated with the correction of mitochondrial function and cell bioenergetics. To this purpose, we examined protein expression levels (Figure 5A), PDH and KGDH activities (Figure 5D,E), iron accumulation (Figure 6A,B), lipoylation levels (Figure 8), mtETC protein expression levels (Figure 9), mitochondrial bioenergetics (Figure 10), and lipid peroxidation (Supplementary Figures S8 and S9) after the supplementation of CocT. All physiopathological alterations in mutant cells were significantly restored. The positive effect of CocT supplementation was also confirmed on iNs obtained by direct reprogramming. Our results showed that CocT treatment increased protein lipoylation levels (Figure 16) and reduced iron overload in mutant *LIPT1* iNs (Figure 17).

Then, we addressed the mechanisms underlying the positive effect of CocT by exploring the activation of sirtuins and therefore their participation in the mtUPR and improving mitochondrial function.

Sirtuins (SIRTs), or NAD^+^-dependent histone deacetylases, are proteins whose deacetylase activity affects the acetylation status of many proteins in the mitochondrial proteome [71]. Moreover, they participate in the regulation of important metabolic pathways in prokaryotes and eukaryotes, such as cell survival, senescence, proliferation, apoptosis, DNA repair, cell metabolism, and caloric restriction [72]. In mammalian cells, there are seven homologs (SIRT1-7) that are distributed in the nucleus (SIRT1, SIRT6, and SIRT7), cytoplasm (SIRT2), and mitochondria (SIRT3, SIRT4, and SIRT5). Reduced sirtuin activity could be a major factor in type 2 diabetes [73], insulin resistance [74], aging [75], cardiopathies [76], mitochondrial diseases [77], neurodegeneration [78], and even antimycobacterial defenses [79]. Although exogenous α-LA cannot be incorporated for protein lipoylation, there is evidence that α-LA supplementation promotes SIRT activation. Thus, Chen W. et al. demonstrated that α-LA supplementation increased SIRT1 activation and the NAD^+^/NADH ratio [80]. They also observed that α-LA upregulated fatty acid β-oxidation and promoted lipid catabolism through the SIRT1/AMP-activated protein kinase (AMPK) signaling pathway.

Sirtuins’ function is highly correlated with NAD^+^, the concentration of nicotinamide, and the activity of nicotinamide phosphoribosyltransferase (NAMPT), which participates in the NAD^+^ biosynthesis. Indeed, research has demonstrated that deficiencies in preserving NAD^+^ levels and the corresponding decrease in sirtuin activity could potentially contribute to the normal aging process [81]. While the use of NAD^+^ precursors, like nicotinamide, has been suggested as a possible complementary agent in numerous treatments, it is unclear if the decline in NAD^+^ could be the cause of the reduced activity of sirtuins [75] or whether, regardless of NAD^+^ concentration, sirtuin expression is reduced with age and disease [82]. The decrease in NAD^+^ may be attributed to impairments in NAMPT-mediated NAD^+^ biosynthesis and PARP-mediated NAD^+^ depletion, which are known pathological processes in aging and potentially in neurodegenerative and mitochondrial disorders [83].

In this study, we found that the SIRT3 activity (Figure 15A) and NAD^+^/NADH ratio (Figure 15E) were impaired in mutant *LIPT1* cells. Interestingly, after CocT treatment, both parameters were significantly increased. Previous studies of our group [63] and other authors [84,85] showed that SIRT3 activation, in combination with mitochondrial cofactors, could boost antioxidant mechanisms, regulate mitochondrial protein quality control, and adapt the OXPHOS system to compensate the pathological consequences of mitochondrial mutations.

SIRT3 is one of the most important deacetylases in mitochondria, and it plays an important role in regulating mitochondrial function [86,87]. For instance, the deacetylation of the PDH complex by SIRT3 enables pyruvate to take part in the Krebs cycle and speeds up the absorption of glucose by triggering protein kinase B (Akt) [88,89]. Additionally, by deacetylating acetyl-CoA synthetase 2 (AceCS2) and long-chain acyl-CoA dehydrogenase (LCAD), SIRT3 guarantees the normalization of fatty acid β-oxidation [90,91,92]. Furthermore, through the deacetylation of 3-hydroxy-3-methylglutaryl-CoA synthetase (HMGCS2), it contributes to the formation of ketone bodies [90,93]. Additionally, the deacetylation of glutamate dehydrogenase (GDH) by SIRT3 induces the utilization of amino acids [94]. Furthermore, ornithine carbamoyltransferase (OTC), a crucial urea cycle enzyme, is a substrate of SIRT3 [95]. Additionally, by deacetylating isocitrate dehydrogenase (IDH) and succinate dehydrogenase (SDH), SIRT3 contributes significantly to the normal progression of the TCA cycle [96,97]. Moreover, the deacetylation of multiple complex I–V subunits within the mtETC suggests that this enzyme plays a crucial role in mitochondrial function [98,99,100]. By activating numerous antioxidant factors, such as FOXO3A, IDH2, and MnSOD, SIRT3 also reduces or delays the damage caused by oxidative stress [101,102,103], thus improving mitochondrial dysfunction and recovering mitochondrial fitness.

In addition, sirtuin activation may induce mitochondrial biogenesis via promoting PGC-1α expression by SIRT3 and PGC-1α deacetylation by SIRT1 [104]. Recent studies have also shown that SIRT3 has a role in mitochondrial quality control, including the refolding or degradation of misfolded/unfolded proteins, mitochondrial dynamics, mitophagy, and mitochondrial biogenesis [87].

Our results indicate that SIRT3 activation is essential for the beneficial effects of the CocT supplementation because its inhibition by 3-TYP, a specific SIRT3 inhibitor, blocked the favorable effect of the treatment on the galactose medium survival or protein lipoylation in mutant cells (Figure 15F, Supplementary Figures S11 and S12).

With these data, we propose that CocT may exert a multi-target function to correct the different pathological processes: first, nicotinamide supplementation may induce the recovery of the NAD^+^/NADH ratio and promotes sirtuin activity; second, α-LA may activate sirtuins and induce the expression of antioxidant enzymes; and third, the rest of the compounds (biotin, vitamin E, thiamine, and pantothenate) may be helpful for correcting the functioning of Krebs cycle enzymes and endogenous α-LA precursors and may increase antioxidant properties in cell membranes. In addition, CocT supplementation was also able to increase the expression levels of the mutant LIPT1 enzyme, which, although dysfunctional, may have some residual activity sufficient to improve the mutant phenotype.

There are several limitations in this study: (1) Only one patient has been included in this work due to the low prevalence of these mutations. (2) Acetylation/deacetylation assays of SIRT3 targets were not analyzed. (3) SIRT3 inhibition by 3-TYP may have “off-target” effects since it has been described that it may also affect methionine aminopeptidase 2 (MetAP2) [105], indoleamine 2,3-dioxygenase 1 (IDO1) [106], and NAD^+^-dependent enzymes, including dehydrogenases [107]. Therefore, it would be of interest to address if genetic SIRT3 depletion would prevent the therapeutic effect of CocT. (4) Further research is needed to determine the molecular mechanism underlying the potential SIRT3 effect in response to CocT. It is possible that *LIPT1* gene mutation may lead to an increased acetylation of mitochondrial protein lysine residues; however, some of these lysine residues/proteins require lipoylation for enzymatic activity (i.e., PDH or α-KGDH). For this reason, it is plausible that the acetylation of these enzymes prevents their lipoylation, leading to the loss of their activity. Therefore, the drug cocktail (CocT) may not only restore the lipoic acid level but also “deacetylate” mitochondrial enzymes for their lipoylation, which may explain why simple lipoic acid supplementation is not effective.

## 5. Conclusions

In our work, we have identified a cocktail of antioxidants and mitochondrial boosting agents able to restore the expression levels of the mutant enzyme, increase the lipoylation of essential mitochondrial proteins, and markedly improve cell bioenergetics in mutant *LIPT1* cells. In addition, the cocktail was able to eliminate iron accumulation and lipid peroxidation associated with a significant improvement in the main physiopathological alterations of mutant *LIPT1* cells. Our data suggest that the positive effect of the cocktail is mediated by SIRT activation, particularly SIRT3, and the expression of antioxidant enzymes, through the induction of mtUPR, an essential protective mechanism in mitochondria. Therefore, the combination of sodium pantothenate, nicotinamide, vitamin E, thiamine, biotin, and α-LA could be of help for correcting *LIPT1* mutations. We have also shown that personalized screenings in cell models derived from patients can be helpful for evaluating the behavior of mutant cells under different therapeutic options and thus identifying the most effective supplements and dose concentrations for their evaluation in controlled clinical trials. In the future, it will be crucial to confirm our findings in 3D (e.g., organoids) and animal models.

## Data Availability

Data and material are available under request.

## Supplementary Materials

The following supporting information can be downloaded at: https://www.mdpi.com/article/10.3390/antiox13081023/s1, Figure S1: Representative images of pharmacological screening in the galactose medium testing the active compounds individually; Figure S2: Representative images of pharmacological screening in the galactose medium testing the combination cocktail (CocT); Figure S3: Quantification of proliferation ratio with different combinations of individual compounds of CocT; Figure S4: The effect of CPI-613, a PDH complex inhibitor, in the survival of control cells; Figure S5: Electron microscopy images of control and patient’s (LIPT1) fibroblasts, both untreated and treated with CocT; Figure S6: Transfection with human LIPT1 plasmid (cDNA) in control and mutant (LIPT1) fibroblasts; Figure S7: Analysis of iron accumulation by cDNA complementation assay; Figure S8: Analysis of mitochondrial membrane peroxidation in both untreated and treated control and mutant (LIPT1) fibroblasts; Figure S9: Analysis of lipid peroxidation in both untreated and treated control and mutant (LIPT1) fibroblasts; Figure S10: Purity of mitochondrial, cytoplasmic, and nuclear fractions; Figure S11: Representative images of pharmacological screening in the galactose medium with 3-TYP, a SIRT3 inhibitor; Figure S12: Protein lipoylation in the presence of 3-TYP, a SIRT3 inhibitor; Figure S13: iNs generated by direct reprogramming from control and patient’s (LIPT1) fibroblasts.

## Abbreviations

3-TYP	3-(1H-1,2,3-triazol-4-yl) pyridine
α-LA	alpha-lipoic acid
α-KGDH	alpha-ketoglutarate dehydrogenase
AceCS2	acetyl-CoA synthetase 2
ACP	acyl carrier protein
ACSM2A	acyl-CoA synthethase medium-chain family member 2A
AMP	adenosin monophosphate
AMPK	AMP-activated protein kinase
*ASCL1*	*achaete-scute family BHLH transcription factor 1*
ATF4	activating transcription factor 4
ATF5	activating transcription factor 5
ATP5F1A	ATP synthase F1 subunit 1 alpha
BCKDH	branched chain ketoacid dehydrogenase
*BRN2*	*POU class 3 homeobox 2*
BSA	bovine serum albumin
CoA	coenzyme A
CoPAN	CoA synthase protein-associated neurodegeneration
COX IV	cytochrome C oxidase subunit IV
CPI-613	6,8-bis (benzylthiol)-octanoic acid
DAPI	4′,6-diamidino-2-phenylindole
DMEM	Dulbecco’s modified Eagle medium
DMSO	dimethyl sulfoxide
FAO	fatty acid oxidation
mtFASII	mitochondrial fatty acid synthesis type II
FBS	fetal bovine serum
FCCP	carbonyl cyanide 4-(trifluoromethoxy) phenylhydrazone
FOXO3A	forkhead box O3
GCS	glycine cleavage system
GCSH	glycine cleavage system H protein
H2AX	H2A histone family member X
HEPES	4-(2-hydroxyethyl)-1-piperazine ethanesulfonic acid
HRP	horseradish peroxidase
Hsp60	heat shock protein 60
Hsp70	heat shock protein 70
IC_50_	half-maximal inhibitory concentration
ICP-MS	inductively coupled plasma mass spectrometry
IDO1	indoleamine 2,3-dioxygenase 1
IEM	inborn error of metabolism
iNs	induced neurons
ISC	iron sulfur cluster
LIAS	lipoic acid synthase
lip3	lipoate-protein ligase 3
*LIPT1*	*lipoyltransferase 1*
LIPT1	lipoyltransferase 1
LIPT2	lipoyl (octanoyl) transferase 2
Lonp1	lon peptidase 1
LplA	lipoate-protein ligase A
MetAP2	methionine aminopeptidase 2
MePAN	mitochondrial enoyl CoA reductase protein-associated neurodegeneration
MnSOD	manganese superoxide dismutase
MRI	magnetic resonance imaging
mt-CO2	mitochondrially encoded cytochrome C oxidase subunit II
mtDNA	mitochondrial DNA
mtETC	mitochondrial electron transport chain
mtUPR	mitochondrial unfolded protein response
NAD^+^	nicotinamide adenine dinucleotide
NAMPT	nicotinamide phosphoribosyltransferase
NBIA	neurodegeneration with brain iron accumulation
nDNA	nuclear DNA
NDUFA9	NADH:ubiquinone oxidoreductase subunit A9
Nrf1	nuclear respiratory factor 1
Nrf2	nuclear respiratory factor 2
OADH	2-oxoadipate dehydrogenase
OCR	oxygen consumption rate
OXPHOS	oxidative phosphorylation
PBS	phosphate-buffered saline
PDH	pyruvate dehydrogenase
PFA	paraformaldehyde
PGC-1α	peroxisome proliferator-activated receptor-γ coactivator 1-α
P-PGC-1α	phosphorylated peroxisome proliferator-activated receptor-γ coactivator 1α
PGK	phosphoglycerate kinase
PKAN	pantothenate kinase-associated neurodegeneration
ROS	reactive oxygen species
SIRT1	sirtuin 1
SIRT3	sirtuin 3
TCA	tricarboxylic acid
TEM	transmission electron microscope
TOMM20	translocase of the outer mitochondrial membrane 20
TFAM	mitochondrial transcription factor A
UQCRC1	ubiquinol cytochrome C reductase core protein 1
VDAC1	voltage-dependent anion channel 1
