# Supplementary material for: A Multi-Target Pharmacological Correction of a Lipoyltransferase LIPT1 Gene Mutation in Patient-Derived Cellular Models
Source: Antioxidants (Basel). 2024 Aug 22;13(8):1023. doi: 10.3390/antiox13081023 (PMC11351668; doi:10.3390/antiox13081023)
Supplement: Supplementary file 1 [file antioxidants-13-01023-s001.zip › antioxidants-3140338-supplementary.pdf]

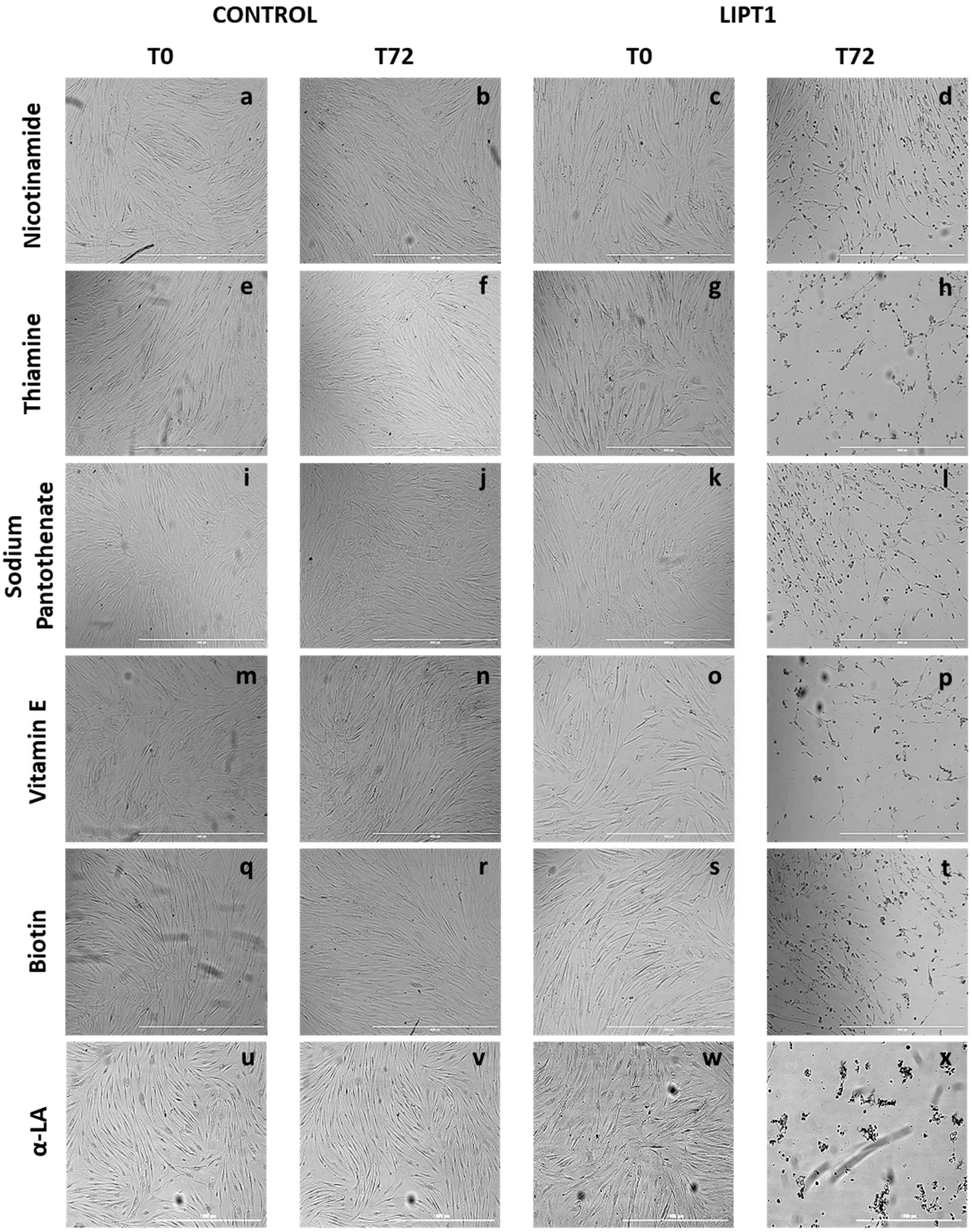

Figure S1: Representative images of pharmacological screening in galactose medium testing the active compounds individually. Control and mutant (LIPT1) **c**ells were seeded in glucose medium and treated with the individual compounds of CocT for seven days. Then, glucose medium was changed to galactose medium, the compounds were renewed and images were taken right now in that moment (T0) and 72hlater (T72). All the compounds acted at 10 µM, except for biotin (5 µM) and sodium pantothenate (4 µM) None of them were able to avoid cell death of patient's fibroblasts in galactose medium (**d, h, l, p, t, x**). Images and cell counting were acquired using the BioTek Cytation 1 Cell Imaging Multi-Mode Reader. Scale bar: 1000 µm.

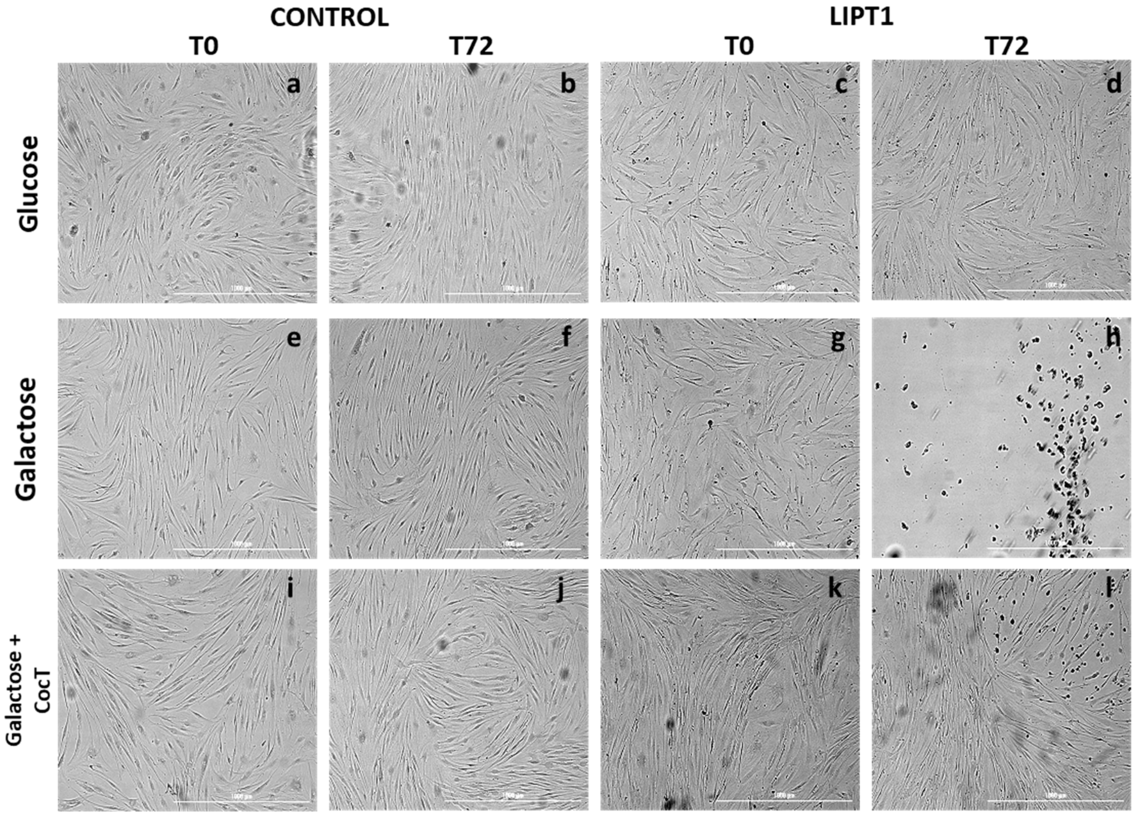

Figure S2: Representative images of pharmacological screening in galactose medium testing the combination cocktail (CocT). Control and mutant (LIPT1) fibroblasts. Cells were seeded in glucose medium and treated with CocT for seven days. Then, glucose medium was changed to galactose medium, the treatment was renewed, and images were taken right now at that moment (T0) and 72h later (T72). In glucose medium, control and mutant *LIPT1* cells presented normal cell proliferation ratio (**b, d**); in galactose medium control cells grew in a normal way (**f**). However, patient's cells died after 72h in galactose medium (**h**). CocT supplementation did not affect proliferation ratio of control cells (**j**). Surprisingly, CocT supplementation enabled the survival of mutant cells in galactose medium (**l**). Images and cell counting were acquired using the BioTek Cytation 1 Cell Imaging Multi-Mode Reader. Scale bar: 1000 µm

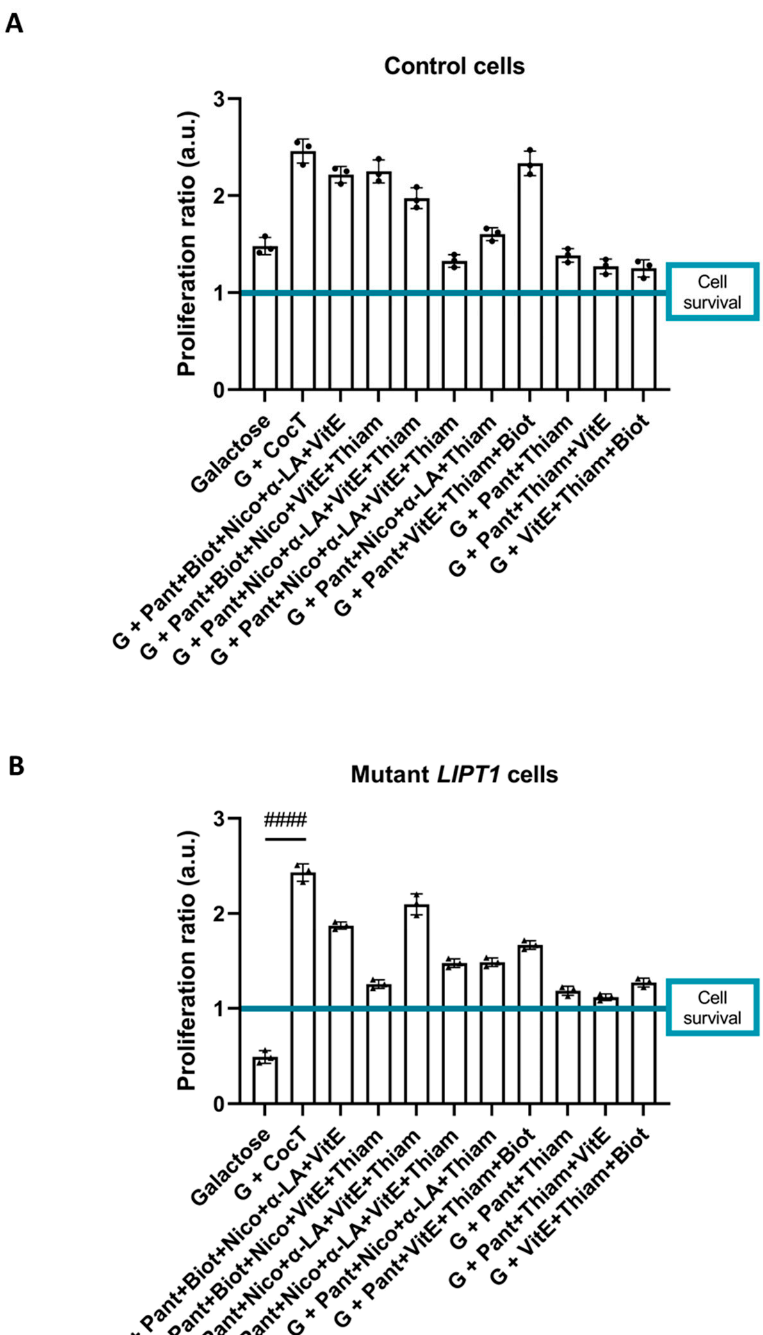

Figure S3: Quantification of proliferation ratio with different combinations of individual compounds of CocT. Control (**A**) and mutant *LIPT1* cells (**B**) were seeded in glucose medium and treated with CocT and different combinations of the individual compounds for seven days. Then, glucose medium was changed to galactose medium and compounds were renewed. G: galactose medium; Pant: sodium pantothenate; Biot: biotin; Nico: Nicotinamide; VitE: vitamin E; Thiam: thiamine; α-LA: α-lipoic acid. All the compounds acted at 10 µM, except for biotin (at 5 µM) and sodium pantothenate (at 4 µM). Cell survival was determined by proliferation ratio (number of cells in T72/ number of cells in T0): >1: cell proliferation ; =1:number of cells unchanged; <1: cell death. ****p<0.0001 between mutant *LIPT1* cells untreated and CocT-treated in galactose medium. a.u.: arbitrary units.

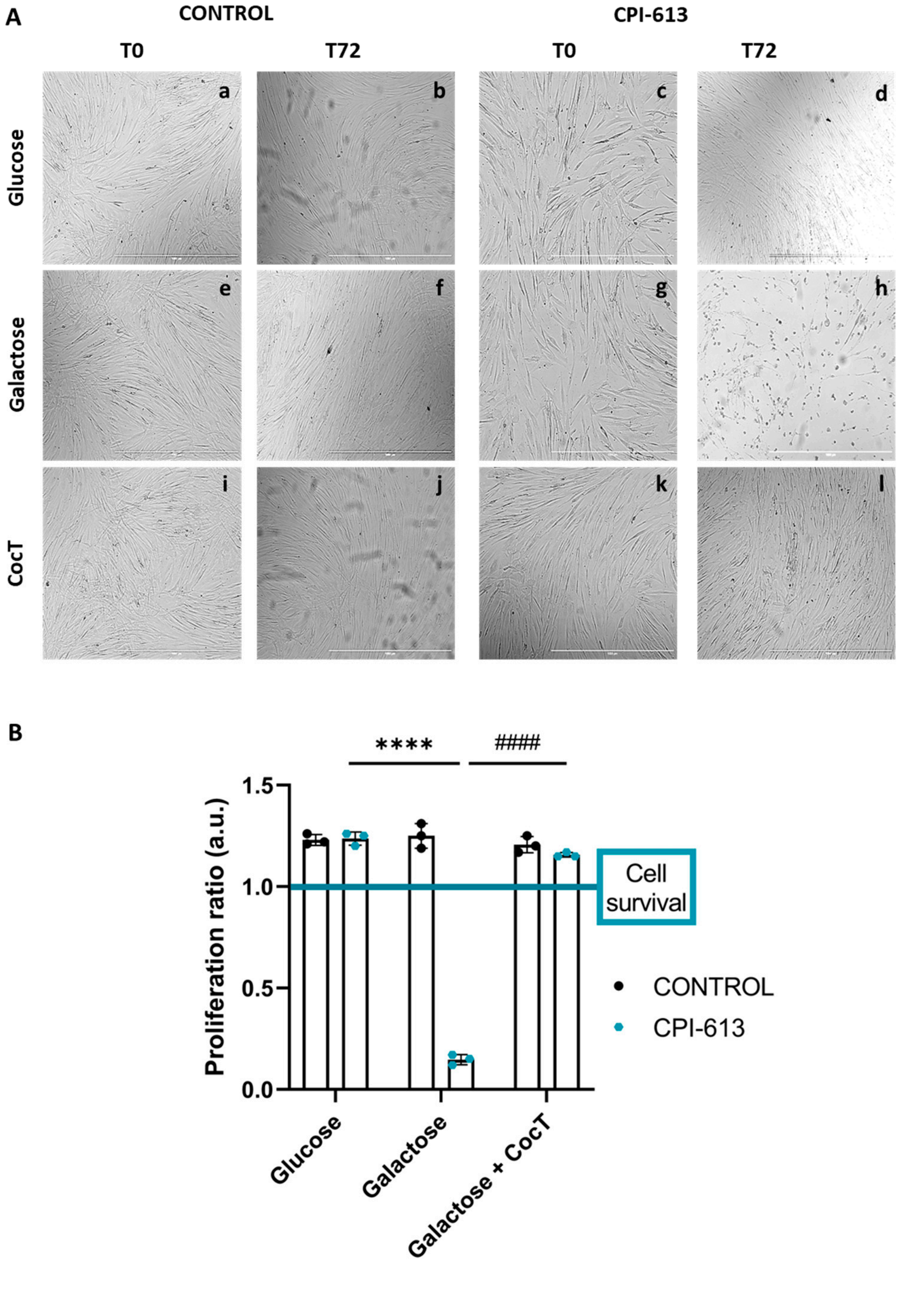

Figure S4: Effect of CPI-613, a PDH complex inhibitor, in the survival of control cells. A. In glucose medium, control cells untreated and treated with CPI-613 were able to grow (**b**, **d**). However, the change to galactose medium did not allow the survival of control cells treated with CPI-613 (**h**) in comparison to untreated control fibroblasts (**f**). The supplementation with CocT restored the growth of cells treated with CPI-613 in galactose medium (**l**). Scale Bar: 1000 µm. **B.** Quantification of the cell survival by proliferation ratio (number of cells in T72/number of cells in T0) in both untreated and treated control fibroblasts. >1: cell proliferation; =1: number of cells unchanged; <1: cell death. ****$p<0.0001$ between CPI-613-treated control cells in glucose medium and galactose medium; ####$p<0.0001$ between CPI-613-treated control cells and CocT-treated control cells in galactose medium. a.u.: arbitrary units.

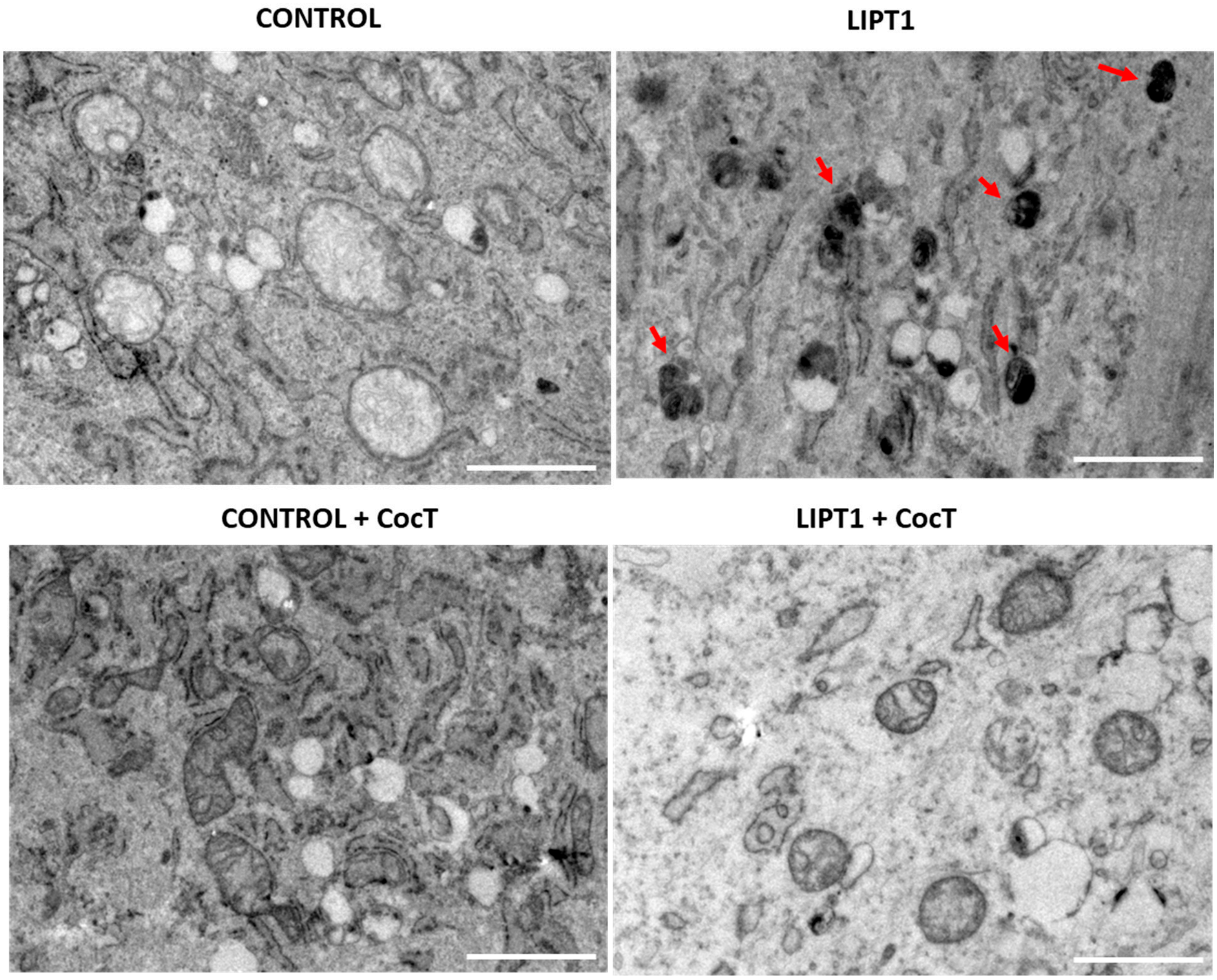

Figure S5: Electron microscopy images of control and patient's fibroblasts (LIPT1), both untreated and treated with CocT. Cells were treated with CocT for seven days. Representative electron microscopy images. Scale bar: 2 µm. Red arrows: lipofuscin-like granules.

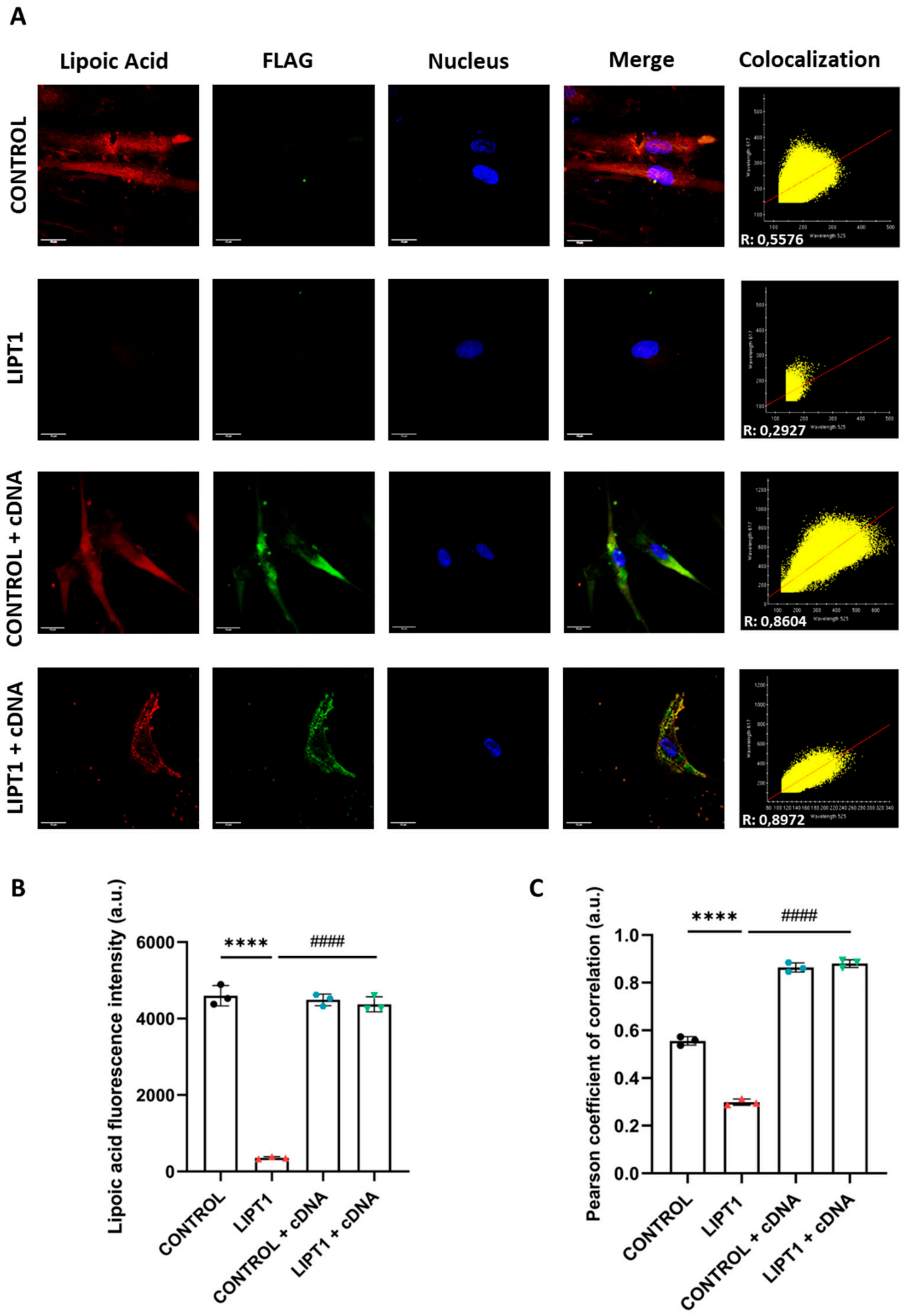

Figure S6: Transfection with human LIPT1 plasmid (cDNA) in control and mutant (LIPT1) fibroblasts. Cells were fixed and inmunostained with anti-lipoic acid and anti-DYKDDDDK tag (FLAG) antibodies **A.** Representative images were acquired DeltaVision microscope. DAPI was used to stain nuclei. Scale bar: 15 µm. **B.** Quantification of fluorescence intensity of lipoic acid antibody. Images were analyzed by ImageJ software (at least thirty images were analyzed per condition). **C.** The colocalization between lipoic acid and FLAG signals was analyzed by Pearson correlation coeficient. Pearson correlation coeficient was calculated by DeltaVision system (at least 30 images were taken and analyzed from each condition and experiment). ****p<0.0001 between control and mutant *LIPT1* fibroblasts. $^{\#\#\#\#}$p<0.0001 between mutant *LIPT1* fibroblasts with and without cDNA complementation. a.u.: arbitrary units.

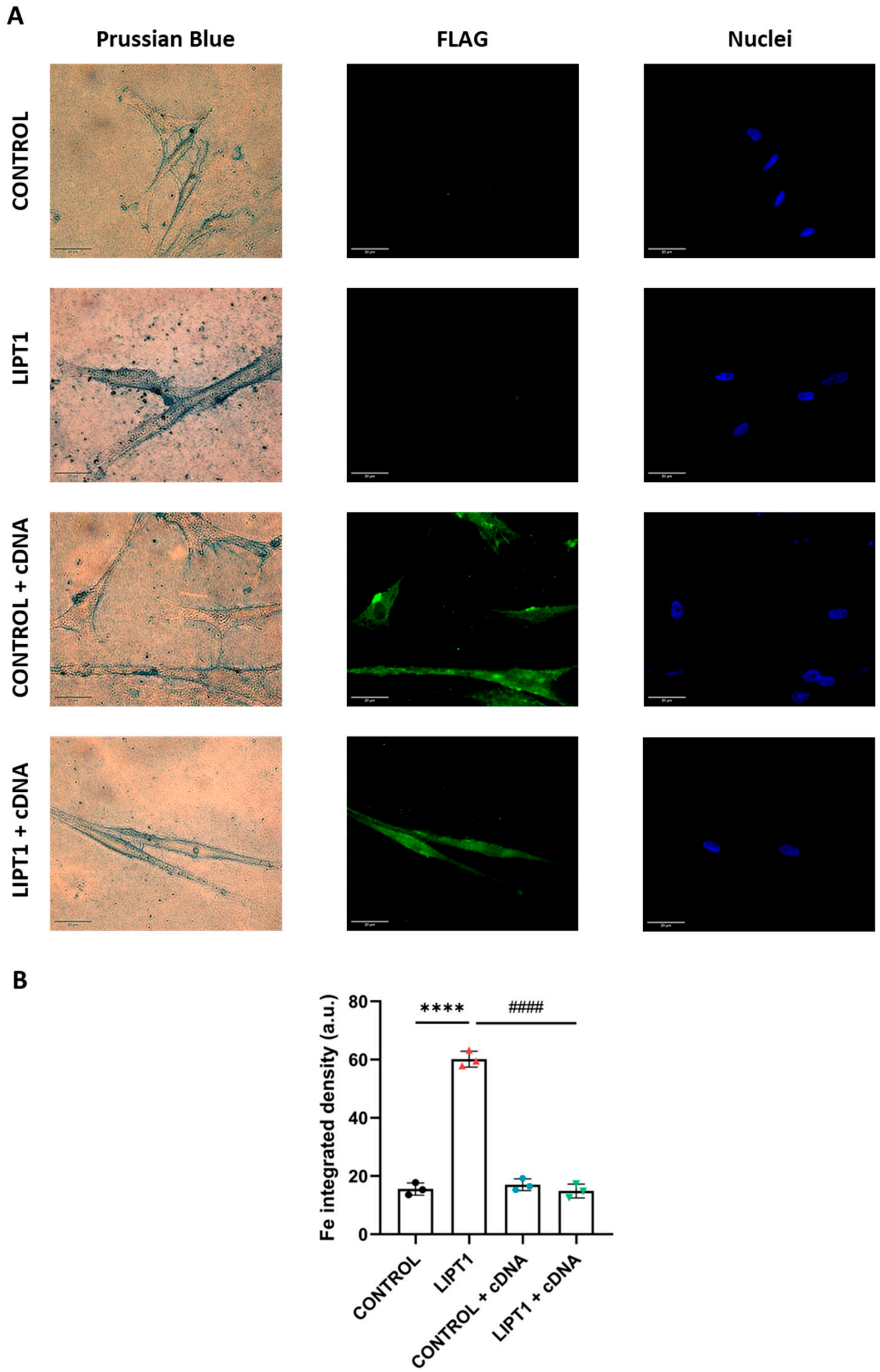

Figure S7: Analysis of iron accumulation by cDNA complementation assay. A. Control and patient's (LIPT1) cells were stained with Prussian Blue staining. Images were acquired by Zeiss Axio Vert V1 microscope. Scale bar: 20 µm. **B.** Quantification of Prussian Blue staining integrated density. Images were analyzed by the ImageJ software (at least 30 images were analyzed per each condition and experiment). Data represent the mean±SD of 3 independent experiments. ****$p<0.0001$ between control and patient's fibroblasts. $^{####}p<0.001$ between mutant fibroblasts with and without cDNA complementation. a.u.: arbitrary units.

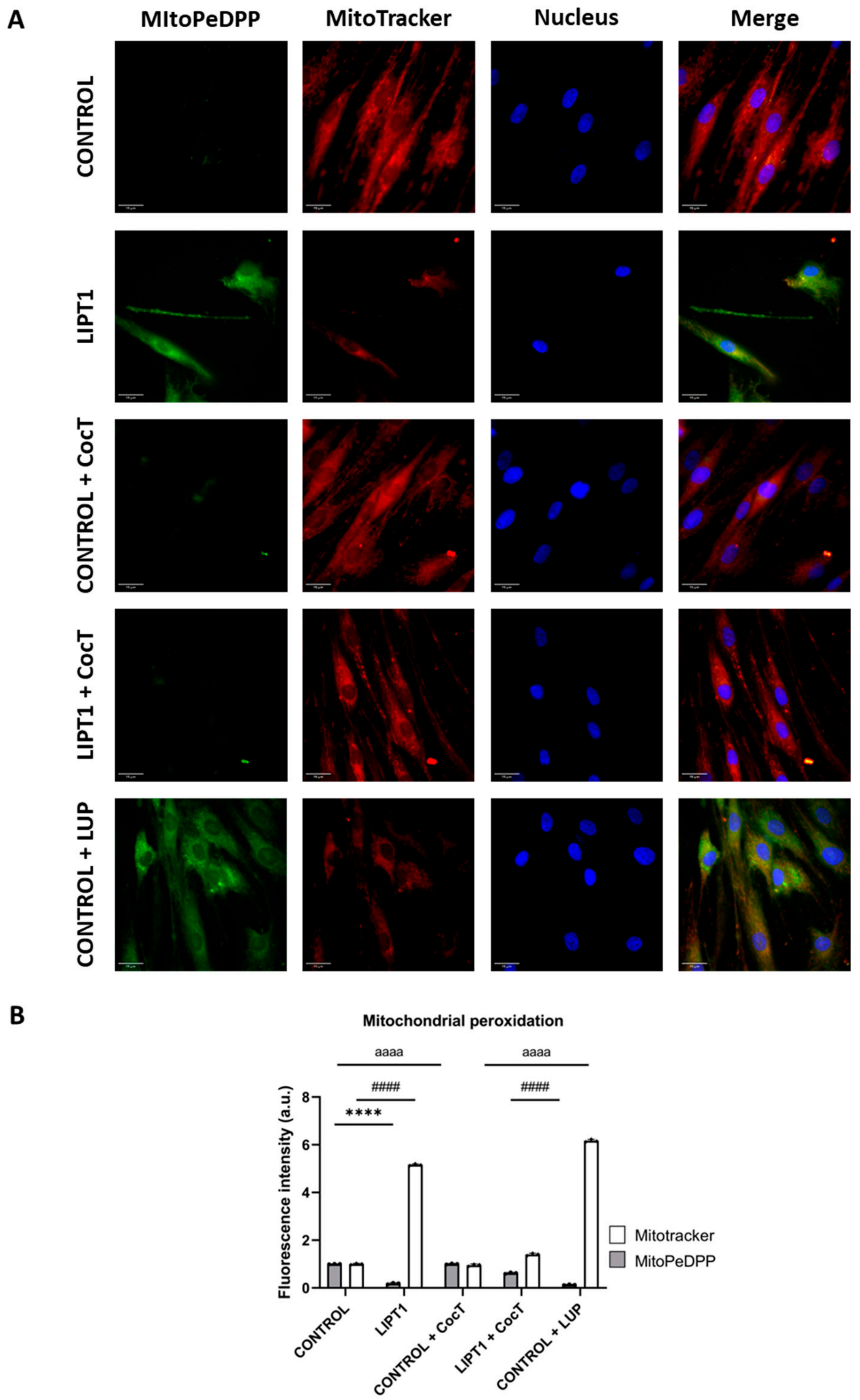

Figure S8: Analysis of mitochondrial membrane peroxidation in both untreated and treated control and mutant (LIPT1) fibroblasts. Cells were treated with CocT for seven days. **A.** Representative images of mitochondrial lipid peroxidation by MitoPeDPP staining. Previously, cells were stained with MitoTracker CMXRos for 45 min. Positive control of mitochondrial peroxidation was included as control cells treated with Luperox at 500 µM (CONTROL + LUP). Scale bar: 15 µm. **B.** Quantification of fluorescence intensity.). Data represent the mean±SD of 3 independent experiments. ****$p<0.0001$ between control and mutant cells. $^{\#\#\#\#}p<0.0001$ between untreated and treated mutant cells. $^{aaaa}p<0.0001$ between untreated and treated control cells with Luperox. a.u.: arbitrary units.

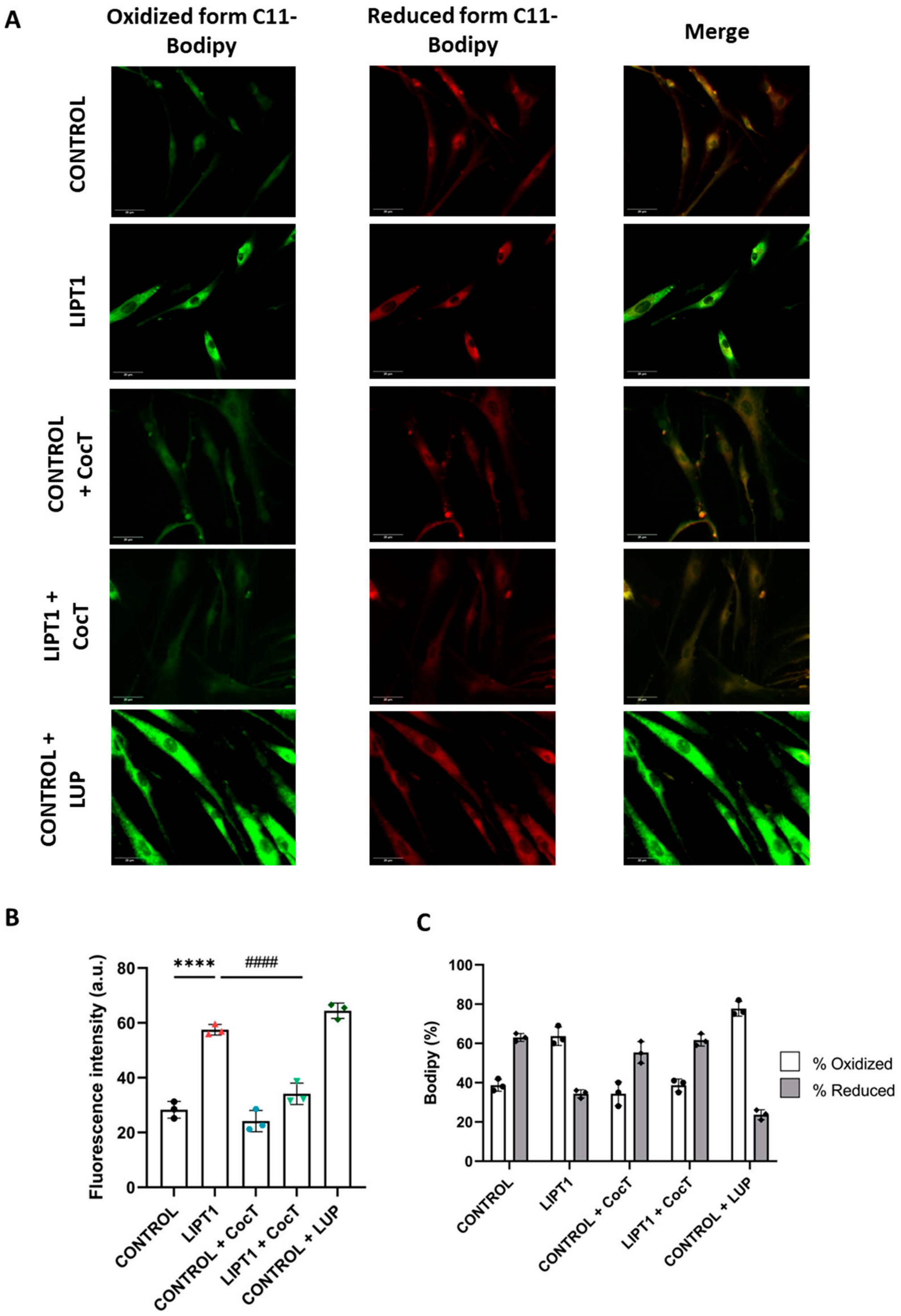

Figure S9: Analysis of lipid peroxidation in both untreated and treated control and mutant (LIPT1) fibroblasts. Cells were treated with CocT for seven days. **A.** Representative images of lipid peroxidation with BODIPY 581/591 C11 staining. Positive control of lipid peroxidation was included as control cells treated with Luperox at 500 µm (CONTROL + LUP). Scale bar: 20 µm. **B.** Quantification of fluorescence intensity. **C.** Representation of the oxidized and reduced fractions. Data represent the mean±SD of 3 independent experiments. ****$p<0.0001$ between control and mutant *LIPT1* fibroblasts. $^{\#\#\#\#}p<0.0001$ between untreated and treated mutant *LIPT1* fibroblasts. a.u.: arbitrary units.

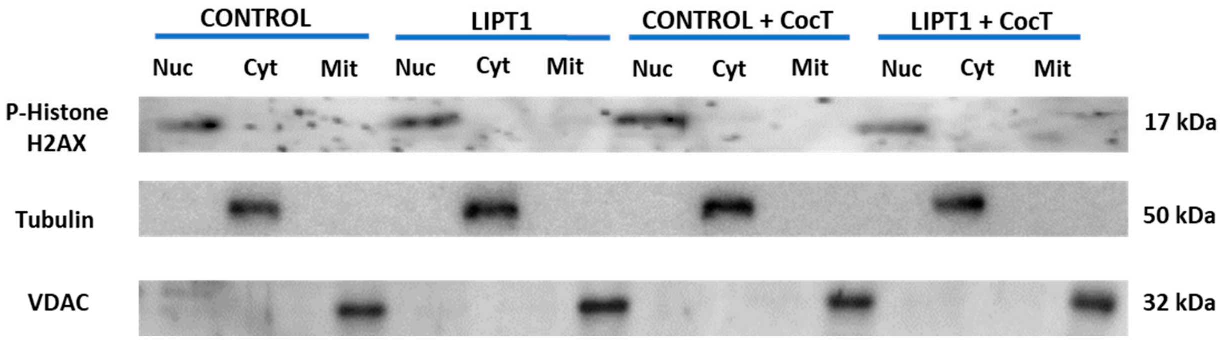

Figure S10: Purity of mitochondrial, cytoplasmic and nuclear fractions. Mitochondrial, cytoplasmic and nuclear fractions were isolated using the Mitochondrial Isolation Kit for Cultured Cells. Data represent the mean±SD of 3 independent experiments.

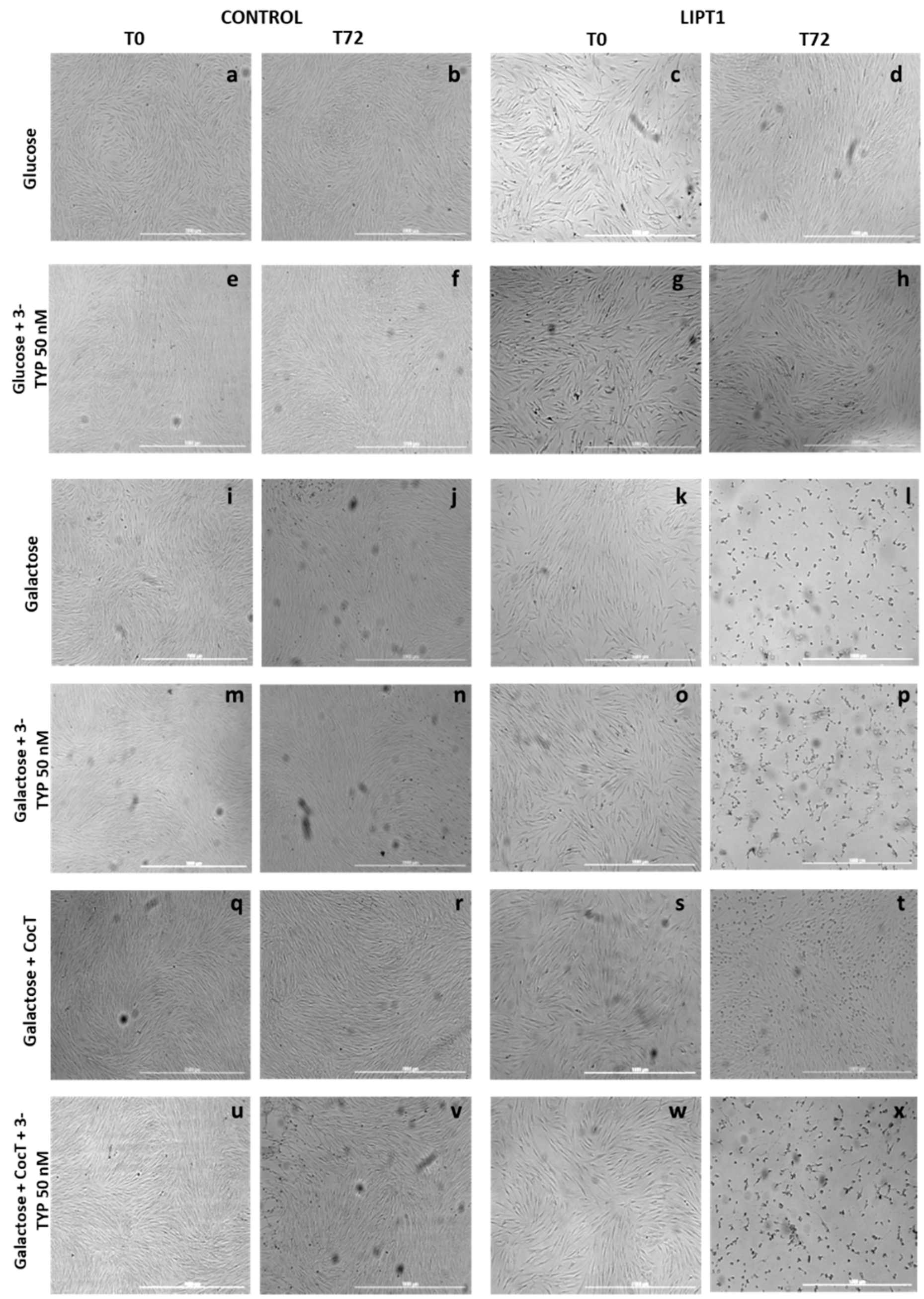

Figure S11: Representative images of pharmacological screening in galactose medium with 3-TYP, a SIRT3 inhibitor. Control and patient's (LIPT1) cells were seeded in glucose medium and treated with CocT for seven days along with 50 nM 3-TYP (added the last 72h of the treatment). Then, glucose medium was changed to galactose medium, treatment was refreshed, and photos were taken right now at that moment (T0) and 72h later (T72). Control cells grew in a normal way in all the conditions galactose medium (**b, f, j, n, r, v**). Patient's cells did not survive in galactose medium (**l**). Nevertheless, they survived with the supplementation of CocT (**t**). 3-TYP blocked the beneficial effect of CocT in mutant *LIPT1* cells (**x**) Proliferation ratio was calculated as number of cells in T72 divided by number of cells in T0, in both control and mutant cells (values > 1: cell proliferation; values = 1: number of cells unchanged; values < 1: cell death). Data represent the mean±SD of 3 independent experiments. Scale bar: 1000 µm

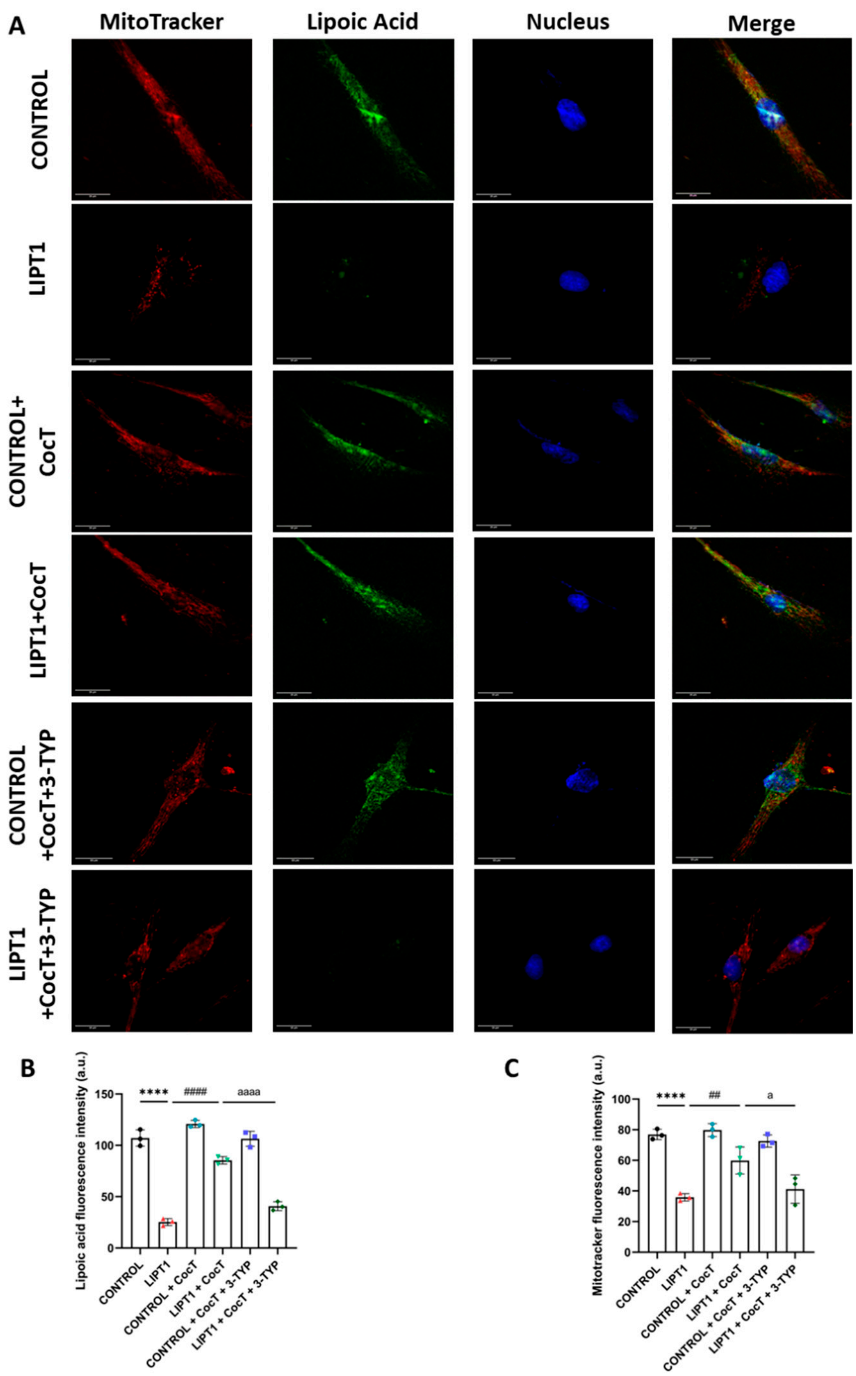

Figure S12: Protein lipoylation in the presence of 3-TYP, a SIRT3 inhibitor. Immunofluorescence assay was performed in both untreated and treated control and mutant (LIPT1) fibroblasts. Cells were treated with CocT for seven days along with 3-TYP (added the last 72h of the treatment). A. Treated and untreated cells were fixed and immunostained with anti-LA antiboy. Mitochondrial network was assessed by MitoTrackerTM Red CMXRos staining. Nuclei were visualized with DAPI staining. Scale bar: 20 µm. B. Quantification of fluorescence intensity of lipoic acid antibody. C. Quantification of fluorescence intensity of MitotrackerTM Red CMXRos. Images were analyzed by Image J software (at least 30 images were analyzed per each condition and experiment). Data represent mean±SD of 3 independent experiments. ****$p<0.0001$ between control and mutant LIPT1 fibroblasts. $^{\#\#}p<0.01$ and $^{\#\#\#\#}p<0.0001$ between untreated and treated mutant LIPT1 fibroblasts. $^{a}p<0.05$ between treated mutant fibroblasts without and with the addition of 3-TYP. a.u.: arbitrary units.

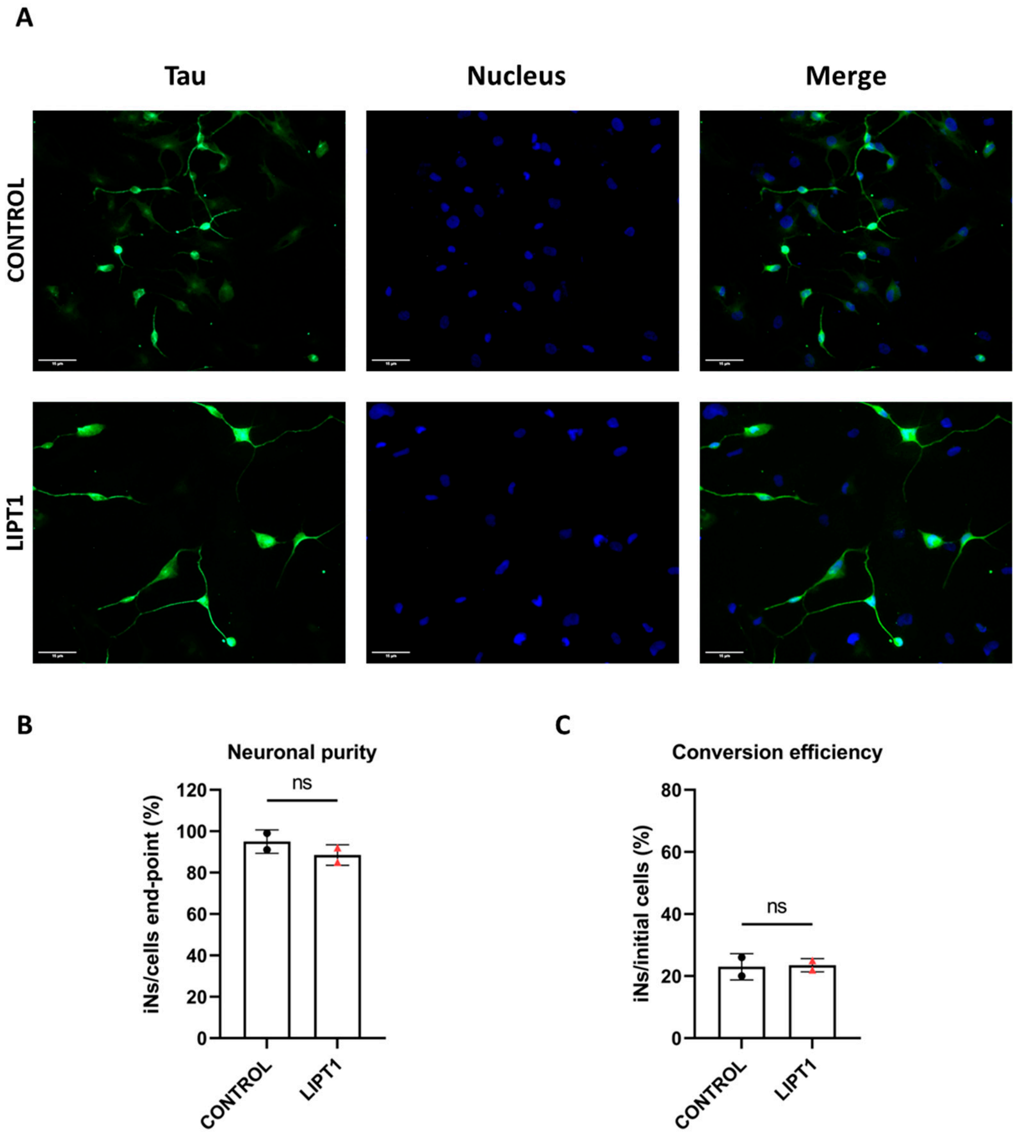

Figure S13: iNs generated by direct reprogramming from control and patient's (LIPT1) fibroblasts. A. Representative images of control and patient's iNs stained against Tau, a neuronal marker. Undifferentiated cells showed only DAPI staining for the nuclei. Scale bar: 15 µm. **B.** Neuronal purity: number of Tau+ cells over the total of cells after reprogramming. **C.** Conversion efficiency: number of Tau+ cells over the total of cells seeded at the beginning of the assay. ns: not significant.
